# Synthesis of inventive biphenyl and azabiphenyl derivatives as potential insecticidal agents against the cotton leafworm, *Spodoptera littoralis*

**DOI:** 10.1186/s13065-023-01050-w

**Published:** 2023-10-27

**Authors:** Eslam A. Ghaith, Hajar A. Ali, Mohamed A. Ismail, Abd El-Aziz S. Fouda, M. Abd El Salam

**Affiliations:** 1 Present Address: Chemistry Department, Faculty of Science, Mansoura University, Mansoura, 35516 Egypt; 2 Plant Protection Research Institute, ARC, Dokki, Giza, Egypt

**Keywords:** Biphenyl, Picolinamidine, Spodoptera littoralis, DFT studies

## Abstract

The emergence of pest resistance of *Spodoptera littoralis* (order; Lepidoptera, family; Noctuidae) towards the large scale of different classes of insecticides necessitates the development of some new poly-functionalized biphenyl and azabiphenyl with highly anticipated insecticidal bioresponse. Four new biphenyl carboxamidines **4a**–**d** and four aza-analogue picolinamidine derivatives **8a**–**d** were designed and prepared via the treatment of their corresponding carbonitriles with lithium-*bis* trimethylsilylamide [LiN(TMS)_2_], followed by hydrolysis with hydrogen chloride. Furthermore, these compounds were elucidated by spectral data, and their toxicity and insecticidal activity were screened against *Spodoptera littoralis*. Whereby, toxicological and biochemical aspects of the inventively synthesized biphenyl and azabiphenyl derivatives against the cotton leafworm, *Spodoptera littoralis* were inspected. As regards the indomitable LC_50_ and LC_90_ values, biphenyl and aza-analogues **8d**, **8a**, **4b**, and **8b**, revealed the furthermost forceful toxic effects with LC_50_ values of 113.860, 146.265, 216.624, and 289.879 ppm, respectively. Whereby, their LC_90_ values are 1235.108, 1679.044, 2656.296, and 3381.256 ppm, respectively, and toxicity index being 22.31%, 17.36%, 11.72%, and 8.76%, respectively, comparing with the already recommended, methomyl insecticide, lannate 90% SP (LC_50_, 25.396 and LC_90_, 57.860 and toxicity index, 100%). Additionally, electrochemical parameters via DFT studies were carried out for demonstrating and elucidation of structure–activity relationship (SAR) according to highly motived compounds^,^ descriptors, and the in vivo insecticidal activities.

**Graphical Abstract:**

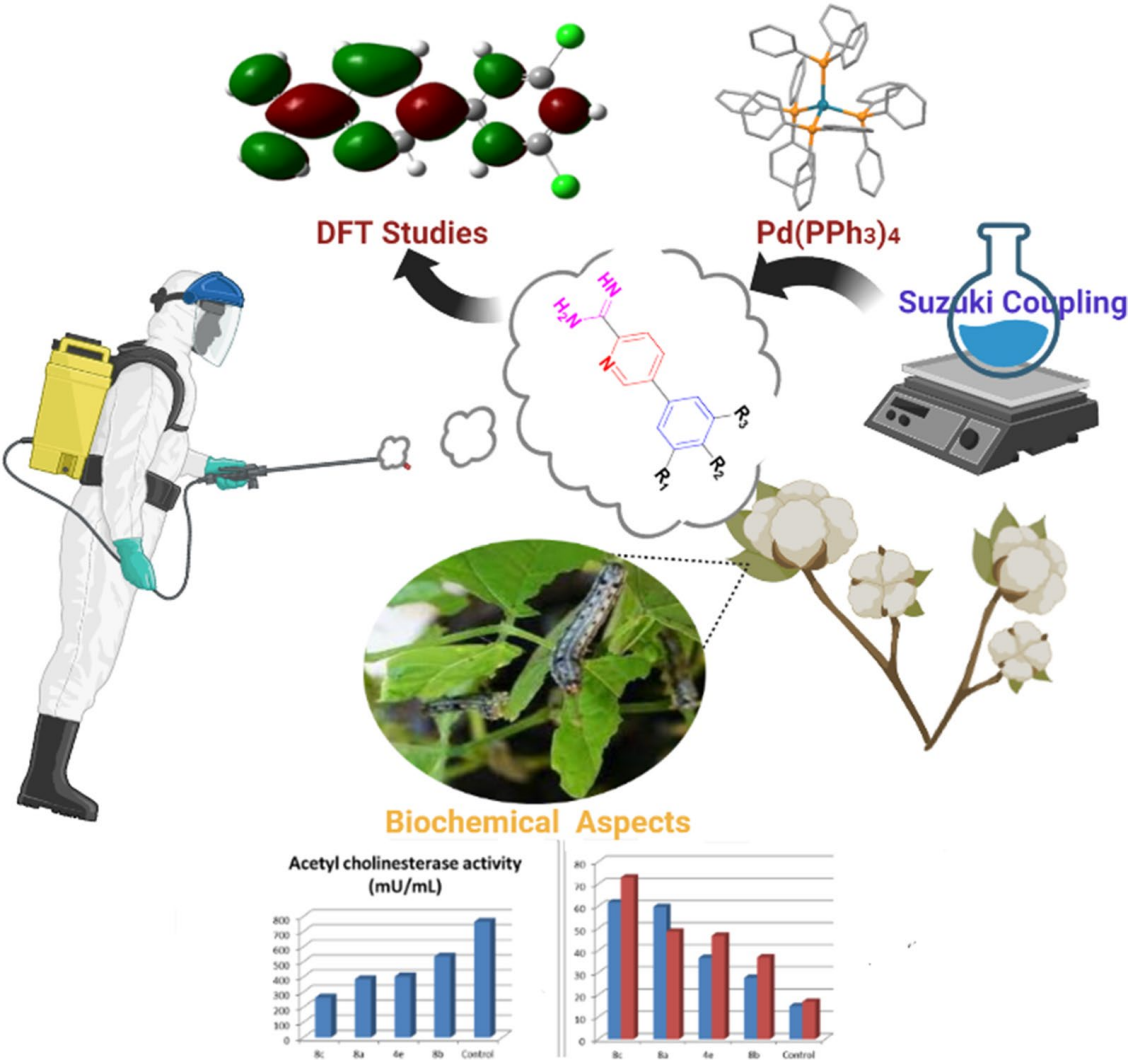

**Supplementary Information:**

The online version contains supplementary material available at 10.1186/s13065-023-01050-w.

## Introduction

Every year, harmful insect pests in agriculture cause considerable losses in crops, and productivity around the world [1–3]. As a result of the incidence of pests, especially animal pests, the growth and productivity of crops become at continuous risk. Despite many measures and developments in crop protection, increasing estimates of actual and potential losses according to Food and Agriculture Organization (FAO) are frequently reported [4–6]. One of the major causes is increased pest resistance to insecticides, which has gradually spread and affected crop growth and harvest [7–10]. Over recent decades, the Egyptian cotton leafworm, *Spodoptera littoralis*, Boisduval (Order; Lepidoptera, Family; Noctuidae) has remained one of the most important and dangerous widespread pests affecting cotton and many important field crops, ornamental plants, vegetables, and weeds in Egypt and many countries over the world [10–12]. As it causes severe damage to these host plants and thus negatively affects the quantity and the quality of agricultural production, subsequently, affecting the national economy of these countries, and increasing the food gap around the world [13–17].

As a result of the increased and excessive use of agricultural pesticides to combat this pest, this led to the emergence of pest resistance, as well as the persistence of residues of these pesticides for long periods, which has adverse effects on humans and beneficial non-target organisms as predators parasites, and thus leads to environmental pollution and disruption of the natural balance [18–20]. For these reasons, we had to build up some inventive bioactive polyfunctionalized biphenyl isosteres of probable insecticidal effectiveness against *S. littoralis* under laboratory circumstances, that are safer for the environment, and less toxic to mammals, without forming cross-resistance to other insecticides [21].

**Fig. 1:**
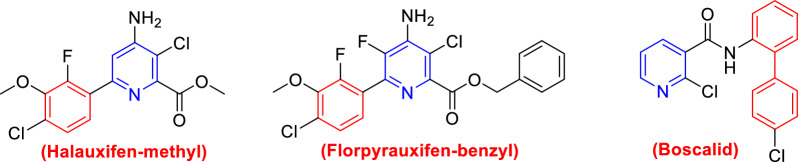
Biologically active aza-biphenyl derivatives

Likewise, assessment of the action of the ultimate potent investigated insecticides by the assessment of enzymatic activities as biochemical aspects for instance, Alk-p, Acid-P, total lipids, lipase activity, total protein, and amylase activity, ALT, AST, and Ach-E. On the other hand, nitrogenous heterocyclic molecules have been playing a crucial role as fungicides, herbicides, insecticides, and plant growth regulators [22–25]. Additionally, compounds containing phenylpyridine derivatives such as halauxifen-methyl, florpyrauxifen-benzyl and boscalid exhibited excellent biological activity against insecticides, fungicides, and herbicides (Fig. 1) [7, 26]. In the same context, the use of halogenated compounds in the development of agrochemical research has significant expansion in the last decade [27, 28]. Encouraged by the aforementioned results, a series of novel biphenylcarboxamidine and their aza-biphenyl analogs substituted with halogens (Cl, F) were designed and synthesized with anticipated pesticidal properties.

## Results and discussion

### Chemistry

**Scheme 1:**
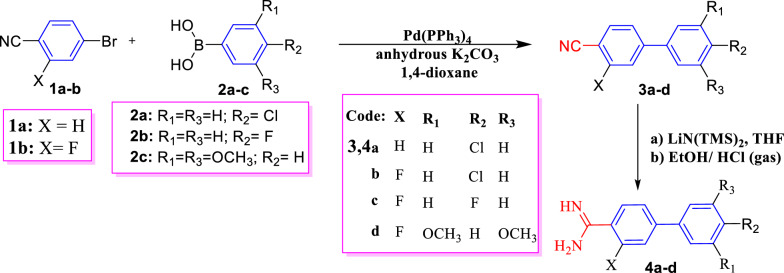
Synthesis scheme for the new biphenyl carboxamidine derivatives **4a**–**d**

A novel mono cationic biphenyl carboxamidine derivatives **4a**–**d** (Scheme 1) were prepared through subsequence steps. Firstly, Suzuki-Miyaura coupling reaction (SMC) of bromobenzonitrile derivatives with substituted phenylboronic acids **2a–c** in the presence of tetrakis(triphenylphosphine)palladium(0) (Pd(PPh_3_)_4_) as a strong electron-donating and sterically bulky catalyst/ligand afforded biphenylnitriles **3a**–**d** in acceptable yield (69–82%) [29]. Secondly, compounds **3a**–**d** were converted to the corresponding free bases of biphenyl carboxamidines **4a**–**d**, on treatment with LiN(TMS)_2_ followed by hydrolysis with hydrogen chloride/EtOH solution. Then, carboxamidine derivatives were neutralized with NaOH to yield the corresponding free bases. Finally, the monoamidines hydrochloride salts **4a**–**d** were synthesized by the reaction of the free base of monoamidines with a mixture of hydrogen chloride/EtOH solution. It is worthy to mention that the attempts to achieve the optimum condition for compounds **3a**–**d** through using different palladium salts palladium(II) carbonate and palladium(II) nitrate (Pd(NO_3_)_2_) led to tedious chromatographic purification and did not impart any increase in yields (50-56%). The spectral analyses of compounds **3a**–**d** were in accordance with their suggested structures. As IR spectra of all mononitriles **3a**–**d** revealed the appearance of the cyano groups in the range of 2225–2231 cm^-1^. Whereby, mass spectra of compound **3a** gave molecular ion peaks m/z at 213.77 (M^+^) and 215.63 (M^+^ +2) due to the presence of chlorine isotopes. ^1^H-NMR spectrum of the biphenylcarbonitrile derivative **3a** displayed four doublet signals resonated at *δ* = 7.54, 7.75, 7.86, and 7.91 ppm with coupling constants of *J* = 9 *Hz* due to four aromatic-H’s of benzonitrile ring and four aromatic-H’s of *para*-chlorophenyl ring, respectively. While in the case of compound **3b**, the presence of fluorine atom in the benzonitrile ring gave a molecular ion peak m/z at 231.15 and 233.15. Whereas a molecular ion peak for compound **3c** appeared at m/z 215.15. ^1^H-NMR spectrum of compound **3d** showed a characteristic singlet signal at *δ* = 3.81 ppm corresponding to *meta*-dimethoxy-H’s. On the other hand, IR spectra of compounds **4a**–**d** confirmed the disappearance of nitrile groups while arising new amidine protons corresponding to NH_2_, ^+^NH_2_ groups. ^1^H-NMR spectrum of compound **4a** confirmed the presence of amidine protons at δ = 9.31, 9.52 ppm and eight aromatic protons; four of them related to *para*-chlorophenyl ring at δ = 7.56 and 7.81 ppm with *J* coupling constant 6.5 *Hz*, whereas the other four protons of benzamidine ring appeared as multiplet signals at *δ* = 7.92–7.96 ppm. The ^13^C-NMR of compound **4a** displayed eight signals corresponding to molecular formula C_13_H_11_ClN_2_, and showed a characteristic signal resonated at *δ* = 165.18 ppm attributed to amidine carbon as showed in supplementary file. In addition, the mass spectrum for chlorobiphenyl amidine **4a** displayed molecular ion peaks at (M^+^, M^+^+2) = 230.82 and 232.46 (44.03, 9.56; chlorine isotopes). The constitution of compound **4b** was affirmed through ^1^H-NMR spectrum which showing two singlet signals corresponding to amidine protons at *δ* = 9.51 and 9.56 ppm, whereby, the aromatic protons corresponding to *para*-chlorophenyl and fluorobenzamidine resonate at *δ* 7.58, 7.75–7.80 and 7.83–7.88 ppm. Whereas, ^19^F-NMR revealed a singlet signal at −*δ*112.57 ppm related to one fluorine atom. In addition, the mass spectrum of **4b** displayed a molecular ion peak at 248.76 compatible with the proposed structure. In the same context, ^1^H-NMR spectrum of compound **4c** demonstrated two multiplet signals of aromatic hydrogens of *para*-fluorophenyl ring at *δ* = 7.33–7.38 and 7.73–7.77 ppm, while the other three aromatic protons of fluorobenzamidine ring appeared at *δ* = 7.83–7.88, besides, the constitute value of singlet signals of cationic amidine protons at *δ* = 9.49 and 9.55 ppm. Furthermore, its mass spectrum displayed that the molecular ion peak of compound **4c**, compatible with the molecular weight at 232.99 corresponding to its molecular formula C_13_H_11_F_2_N_2_^+^.

**Scheme 2:**
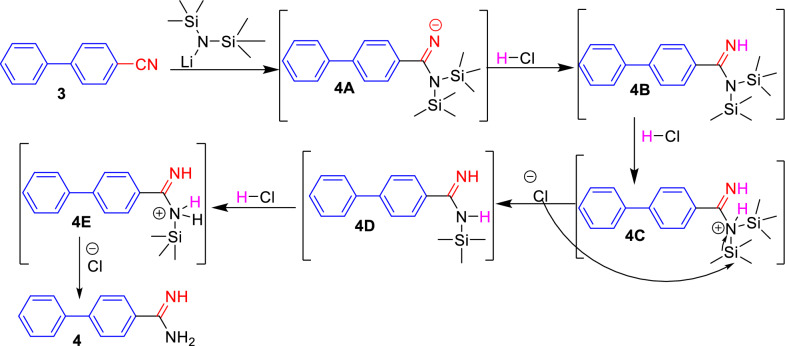
The proposed mechanism of the formation of biphenyl carboxamidine **4**

The formation of carboxamidine derivatives **4** may be suggested on the basis of a mechanism that proceeded through the nucleophilic attack of LiN(TMS)_2_ to nitrile carbon of biphenylcarbonitrile **3** to furnish [1,1ʹ-biphenyl] (bis(trimethylsilyl)amino)methylene)amide **4A** as the mechanism involving the addition of lithium amide to the nitrile group, followed by protonation by desilylation reaction using hydrogen chloride led to the attraction of acidic hydrogen by nitrogenous anion to yield intermediate **4B** [30, 31]. Then, the chloride anion attacks the most positive silicon atom leading to the concurrent expel of trimethylsilyl chloride molecule (CH_3_)_3_SiCl as a volatile liquid. Finally, the previous steps were repeated passing through intermediates **4C** and **4D** till affording carboxamidines **4a**–**d** (Scheme 2).

**Scheme 3:**
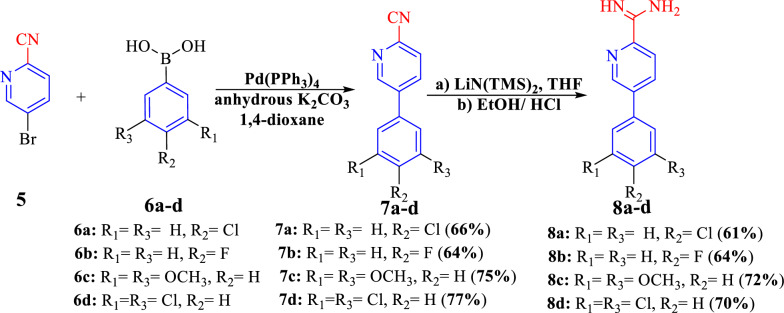
Synthesis of substituted phenylpicolinamidines

On the same manner, treatment of bromopicolinonitrile **5** with substituted-phenylboronic **6a**–**d** in refluxing dioxane and in the presence of Pd(PPh_3_)_4_ and K_2_CO_3_ yielded the corresponding phenylpicolinonitrile derivatives **7a**–**d** (Scheme 3). Then, the transformation of phenylpicolinonitriles to phenylpicolinamidine derivatives **8a**–**d** (Scheme 3) via the same steps mentioned above for synthesis of biphenyl amidine. Spectral analysis of compounds **7a**–**d** and **8a**–**d** were in accordance with the suggested structures. The structures of compounds **7a**–**d** were supported by IR spectra which revealed the presence of nitrile group at 2227 to 2239 cm^−1^. ^1^H-NMR spectrum of compound **7a** manifested two doublet signals at* δ* = 7.61 and 7.88 ppm with the same *J* = 8.4 *Hz* for the aromatic protons of the *para*-chlorophenyl ring, another characteristic doublet signal observed at* δ* = 8.13 ppm with *J* = 8.1 *Hz*, besides, the presence of doublet of doublet signals at 8.36 and a singlet signal at 9.10 corresponding to Ar–H of picolinonitrile ring. Its ^13^C-NMR spectrum showed ten signals and exhibited a characteristic signal at* δ* = 117.57 ppm corresponding to the carbonitrile group. The mass spectrum of picolinonitrile **7a** gave a molecular ion peak for C_12_H_7_ClN_2_ at m/z = 214.10 (M^+^, 85.36%) and 216.15 (27.28%) due to chlorine isotopes. In the ^1^H-NMR spectrum of compound **7c,** *meta*-dimethoxy-H's reverberated as a singlet signal at* δ* = 3.83 ppm and two singlet signals at* δ* = 6.62 and 6.95 ppm for the Ar–H of dimethoxyphenyl ring. The ^13^C-NMR spectrum for compound **7c**, revealed eleven carbon signals that corresponded to the elucidated structure and rebounded at* δ* = 55.48 ppm corresponding to two methoxy groups at meta position and* δ* = 117.65 ppm for the cyano group. The aromatic protons of picolinonitrile ring were observed as two doublet signals at* δ* = 8.10 and 8.35 ppm, in addition to a singlet signal that appeared at* δ* = 9.10 ppm. Moreover, its mass spectrum showed a molecular ion peak at m/z 240.20 (100%), for a molecular formula C_14_H_12_N_2_O_2_. Conversely, the IR spectra of compounds **8a**–**d** confirmed that the nitrile group had vanished, while new bands at 3272–3445 that corresponded to NH_2_, ^+^NH_2,_ and groups had appeared. The ^1^H-NMR spectrum of compound **8a** exhibited the aromatic protons as two doublet signals at* δ* = 7.62 and 7.92 ppm with *J* = 9 *Hz* for *para*-chlorophenyl ring, while the aromatic protons of picolinamidine ring reverberated as two doublet and one singlet signals at* δ* = 8.44, 8.50 and 9.14 ppm, respectively. In addition, two singlet signals at 9.46 (s, 2H, H_2_N, D_2_O exchangeable), and 9.66 ppm (s, 2H, NH_2_^+^, D_2_O exchangeable). Whereas, the ^13^C-NMR spectrum of compound **8a** displayed ten signals and revealed the characteristic carbon signal rebounded at *δ* = 161.58 ppm corresponding to cationic amidine carbon. Its mass spectrum gave the molecular ion at m/z = 231.0 and the base peak at m/z = 63.95. The main characteristic features of ^1^H-NMR spectrum of **8b** showed two singlet signals at* δ* = 9.51 (s, 2H, NH_2_ exchangeable with D_2_O), 9.69 ppm (s, 2H, ^+^NH_2_ exchangeable with D_2_O), in addition two doublet signals related to fluorophenyl at *δ* 7.40 and 7.96 ppm, whereas, aromatic protons of picolinamidine appeared as a multiple signals at *δ* 8.46–8.47 ppm. ^19^F-NMR showed a characteristic singlet signal at −* δ* 112.39 ppm (using TFA as external standard) of fluorine atom. Whereby, its IR spectrum confirmed the disappearance of cyano group while arising a new band at 3411 and 1686 corresponding to NH, C=N, respectively. While, its mass spectrum showed a molecular ion peak at 215.05 which is completely compatible with its molecular weight C_12_H_10_FN_3_. Furthermore, ^1^H-NMR spectrum for compound **8c** exhibited a characteristic singlet signal at *δ* 3.83 assigned to two *methoxy groups*, as well as, two singlets at 6.63 and 6.99 ppm for the aromatic protons of dimethoxyphenyl ring. Aromatic protons of picolinamidine ring were found as two doublets at* δ* = 8.42, 9.13 ppm and one doublet of doublet signal at 8.48 with *J* = 1.5 and 8 *Hz*, besides, two singlet signals at* δ* = 9.45 and 9.65 ppm for amidic protons (H_2_N, NH_2_^+^). A molecular ion peak of compound **8c** was noticed as m/z = 257.10 (M^+^, 39.08%) and while m/z = 241.05 corresponding to its base peak. Also, ^13^C-NMR spectrum assured the presence of eleven signals, whereas, amidine carbon resonated at *δ* =161.61 ppm and two methoxy carbons resonated at 55.50 ppm.

### Insecticidal activity

#### Toxicological activity

##### Toxicity test for Cotton leaf worm (*S. littoralis*, Order; Lepidoptera, Family; Noctuidae)

The represented data in Table 1 demonstrated the insecticidal efficacy of eight innovatively tested compounds against the 2nd instar larvae of the laboratory strain of the polyphagous pest, cotton leafworm, *S. littoralis* (Boisd.) at different concentrations. The represented data displayed that the mortality percentages directly increase with the increasing of concentrations and days post-treatment. Consequently, compounds; **8d**, **8a**, **4b,** and **8b** were the most potent compounds, where, the mortality percentages of the treated larvae of *S. littoralis* reached 86.67%, 83.34%, 76.67% and 70.00%, respectively. The total of inspected compounds displayed powerful toxic effects after 7 days (based on LC_50_ value). Amongst all, compounds **8d**, **8a**, **4b** and **8b** exhibit excellent results with LC_50_’s values 113.860, 146.265, 216.624 and 289.879 ppm, respectively. In the same context, the toxicity indexes are 22.31%, 17.36%, 11.72%, and 8.76%, respectively, compared with the already recommended, methomyl insecticide, lannate 90% SP (LC_50_, 25.396 & LC_90_, 57.860 and toxicity index, 100%), according to the toxicity data denoted in Table 2.

**Table 1:** Mortality% of the 2nd instar larvae of cotton leafworm*, S. littoralis* after treatment by the newly synthesized compounds compared with Methomyl insecticide, Lannate 90% SP after 7 days of treatment

Tested compounds	Concentrations	Mortality % after days post treatment			
		1 day	3 days	5 days	7 days
**Lannate** **90% SP**	**12.5**	3.33	6.67	10.00	13.33
	**25**	26.67	30	36.67	46.67
	**50**	66.67	76.67	83.33	90
	**100**	80.00	83.33	90.00	**96.67**
**8d**	**25**	3.34	10.00	16.67	20.00
	**50**	6.67	13.34	20.00	30.00
	**100**	23.34	33.34	43.34	53.34
	**200**	30.00	40.00	53.34	63.34
	**400**	33.34	43.34	56.67	70.00
	**800**	46.67	63.34	76.67	**86.67**
**8a**	**25**	0	3.34	10.00	16.67
	**50**	0	6.67	16.67	26.67
	**100**	16.67	26.67	36.67	46.67
	**200**	23.34	36.67	46.67	60.00
	**400**	26.67	40.00	53.34	63.34
	**800**	50.00	60.00	73.34	**83.34**
**4b**	**25**	0	0	6.67	10.00
	**50**	0	3.34	13.34	23.34
	**100**	13.34	23.34	36.67	43.34
	**200**	16.67	26.67	40.00	50.00
	**400**	23.34	33.34	43.34	53.34
	**800**	46.67	56.67	66.67	**76.67**
**8b**	**25**	0	0	0	6.67
	**50**	0	0	6.67	16.67
	**100**	6.67	16.67	26.67	36.67
	**200**	13.34	23.34	33.34	43.34
	**400**	16.67	26.67	40.00	50.00
	**800**	36.67	46.67	56.67	**70.00**
**4c**	**25**	0	0	0	3.34
	**50**	0	0	3.34	13.34
	**100**	3.34	6.67	20.00	30.00
	**200**	6.67	16.67	26.67	36.67
	**400**	13.34	23.34	36.67	46.67
	**800**	33.34	43.34	53.34	**63.34**
**4a**	**25**	0	0	0	0
	**50**	0	0	0	6.67
	**100**	0	3.34	13.34	23.34
	**200**	6.67	13.34	20.00	33.34
	**400**	3.34	13.34	23.34	36.67
	**800**	13.34	26.67	43.34	**53.34**
**4d**	**25**	0	0	0	0
	**50**	0	0	0	0
	**100**	0	0	3.34	13.34
	**200**	0	0	6.67	20.00
	**400**	3.34	6.67	20.00	30.00
	**800**	6.67	23.34	33.34	**43.34**
**8c**	**25**	0	0	0	0
	**50**	0	0	0	0
	**100**	0	0	0	6.67
	**200**	0	0	3.34	13.34
	**400**	0	3.34	10.00	16.67
	**800**	3.34	16.67	26.67	**36.67**

Bold represents the optimal concentration exhibiting the highest efficacy of each tested compound as an insecticidal agent after 7 days post treatment

**Table 2:** Toxicity bioresponses of the recently synthesized compounds towards the 2nd instar larvae of cotton leafworm, *S. littoralis* compared with methomyl insecticide, Lannate 90% SP after 7 days post treatment

Tested compounds	LC_50_ (ppm) and confidence limits at 95%	LC_90_ (ppm) and confidence limits at 95%	Slope	Toxicity index % at LC_50_ value	Relative potency
**Lannate** **90% SP**	25.396	57.860	3.584 ± 0.552	100	74.47
	20.725	45.600			
	30.600	85.143			
**8d**	113.860	1235.108	1.238 ± 0.207	22.31	16.61
	75.479	653.341			
	165.348	3990.189			
**8a**	146.265	1679.044	1.209 ± 0.205	17.36	12.93
	98.758	836.464			
	217.972	6226.086			
**4b**	216.624	2656.296	1.177 ± 0.206	11.72	8.73
	147.368	1198.720			
	346.844	12500.646			
**8b**	289.879	3381.256	1.201 ± 0.211	8.76	6.53
	197.005	1467.575			
	492.252	17390.008			
**4c**	400.239	4369.241	1.235 ± 0.220	6.35	4.73
	267.559	1811.834			
	739.401	25266.588			
**4a**	666.646	9419.460	1.114 ± 0.209	3.81	2.84
	437.575	3615.027			
	1249.568	68880.669			
**4d**	1048.448	12951.331	1.174 ± 0.208	2.42	1.81
	710.313	5598.709			
	1758.127	67818.313			
**8c**	1891.266	20255.647	1.245 ± 0.180	1.34	1.00
	1320.721	9886.043			
	2980.333	68954.267			

Toxicity index is demarcated as the ratio of the utmost operative compound’s LC_50_ value to the other examined compound’s LC_50_ value multiplying by 100

#### Biochemical impacts

##### A. Alkaline phosphatase (Alk-P) and Acid phosphatase (Acid-p) analyses

In Table 3, the data presented that **8d** achieved the highest drop in the activity of Alk-P, lower than in the control, it was − 41.99%, followed by **8a**, **4b**, of which, it was by − 35.81, − 30.63%, respectively, while the nethermost decrease in Alk-P activity was prompted by **8b**, by − 26.82% lower than the control sample. Additionally, **8d** revealed the maximum reduction in the activity of Acid-P lower than in the control, it was − 85.10%, followed by **8a**, **4b**, of which, it was by − 81.94, − 78.84%, whereas **8b** caused the lowest drop in Acid-P activity, by − 73.96% less than the control sample.

**Table 3:** The activities of Alk-P and Acid-p in Hemolymph of the 4th Instar Larvae of *S. littoralis* later 5 days of action with LC_50_ of **8d**, **8a**, **4b**, and **8b** tested compounds

Tested compounds	Alkaline phosphatase (U/L)	% of control	Acid phosphatase (U/L)	% of control
**8d**	56.45 ± 0.28^e^	− 41.99	18.37 ± 0.23^e^	− 85.10
**8a**	62.47 ± 0.22^d^	− 35.81	22.26 ± 0.27^d^	− 81.94
**4b**	67.51 ± 0.23^c^	− 30.63	26.08 ± 0.28^d^	− 78.84
**8b**	71.22 ± 0.27^b^	− 26.82	32.10 ± 0.44^b^	− 73.96
Control	97.31 ± 0.22^a^		123.23 ± 0.40^a^	

Whereby, means ± SE (standard error) of 3 replicates of 50 of 4th larvae each. Distinct letters designate significant variances between treatments as stated by Duncan’s test. LSD = 0.05 was 0.769 U/L for ***Alk-P*** and 1.048 U/L for ***Acid-p***. % of control = (test−control)/control × 100

##### B. Total proteins and total lipids activity’s analyses

Looking at Table 4, it can be detected that all of the examined compounds produced a reduction in total proteins; it ranged from − 3.96 to − 73.97% lower than in the control. The activity of the **8d** treated with larvae reached to its lowest level (− 85.07% lower than in the control), and **8b** caused the least salient decrease in the total lipids' activity (− 2.86% compared to the control). The results showed that all of the synthesized compounds tested caused a notable reduction in total lipid activity (Table 4).

**Table 4:** Total proteins and total lipids, activities in the hemolymph of the 4th Instar Larvae of *S. littoralis* later 5 days of treatment with LC_50_ of **8d**, **8a**, **4b**, and **8b** tested compounds

Tested compounds	Total proteins (g/dL)	% of control	Total lipids (g/dL)	% of control
**8d**	5.40 ± 0.31^d^	− 73.97	3.03 ± 0.12^d^	− 85.07
**8a**	8.19 ± 0.27^c^	− 60.52	8.36 ± 0.30^c^	− 58.82
**4b**	14.40 ± 0.31^b^	− 30.57	14.63 ± 0.35^b^	− 27.93
**8b**	19.92 ± 0.20^a^	− 3.96	19.72 ± 0.35^a^	− 2.86
Control	20.74 ± 0.31^a^		20.30 ± 0.28^a^	

Whereby, means ± SE (standard error) of 3 replicates of 50 of 4th larvae each. Distinct letters designate significant variances between treatments as stated by Duncan’s test. LSD = 0.05 was 0.888 g/dL for total proteins and 0.921 g/dL for total lipids. % of control = (test−control)/control × 100

##### C. Determination of lipase and amylase activity

Table 5 depicts the results of lipase and amylase activities for the tested compounds, as compound **8d** caused a significant increase in the lipase activity; it was 47.56% higher than in the control while the other tested compounds **8a**, **4b**, and **8b** showed a significant decrease in lipase activity; it was by − 70.73, − 70.73 and − 53.66%, respectively, lower than in the control but not significant between each other. Conversely, the obtained results demonstrated that all the investigated compounds caused a notable decrease in the amylase activity (Table 5), the activity of **8b** treated larvae fluctuated to its lowest pattern level (− 89.24% lower than in the control), while **8d**, **8a** and **4b** caused remarkable decrease by − 56.06, 65.03 and 81.62%, respectively, lower than in the control.

**Table 5:** Lipase and amylase activities in Hemolymph of the 4th Instar Larvae of *S. littoralis* after 5 days of exposure with LC_50_ of **8d**, **8a**, **4b**, and **8b** tested compounds

Tested compounds	Lipase activity (U/mL)	% of control	Amylase activity (U/mL)	% of control
**8d**	0.121 ± 0.004^a^	47.56	0.098 ± 0.002^b^	− 56.06
**8a**	0.024 ± 0.007^c^	− 70.73	0.078 ± 0.007^b^	− 65.03
**4b**	0.024 ± 0.002^c^	− 70.73	0.041 ± 0.002^c^	− 81.62
**8b**	0.038 ± 0.002^c^	− 53.66	0.024 ± 0.003^c^	− 89.24
Control	0.082 ± 0.002^b^		0.223 ± 0.015^a^	

Whereby, means ± SE (standard error) of 3 replicates of 50 of 4th larvae each. Distinct letters designate significant variances between treatments as stated by Duncan’s test. LSD = 0.05 was 0.038 U/mL for lipase and 0.023 U/mL for amylase. % of control = (test−control)/control × 100

##### D. Alanine aminotranferase (ALT) and asparate aminotransferase (AST) activities, analyses

The data showed that all the established compounds displayed a noteworthy accretion in ALT activity (Table 6), and the enzyme activity reached its ultimate value in **8d** treated larvae (313.18%). Whereas the enzyme activity was observed to be at the lowermost increase in **8b** treated larvae (85.09%). Compounds **8a** and **4b** have remarkable increases in enzyme activity by 299.00% and 145.56%, respectively, higher than the control. In addition, there was a rise in the activity of asparate aminotransferase (AST) (Table 6). Whereas, compound **8d** was the highest potent compound having insecticidal properties, as it displayed a vastly significant enzyme activity exceeding the control sample (330.78%), followed by **8a** (187.07), **4b** (175.55%) then **8b** (118.14%).

**Table 6:** Changes of ALT and AST activities in Hemolymph of the 4th Instar Larvae of *S. littoralis* later 5 days of treatment with LC_50_ of **8d**, **8a**, **4b**, and **8b** tested compounds

Tested compounds	ALT activity (U/L)	% of control	AST activity (U/L)	% of control
**8d**	61.77 ± 0.32^a^	313.18	72.93 ± 0.53^a^	330.78
**8a**	59.65 ± 0.47^b^	299.00	48.60 ± 0.49^b^	187.07
**4b**	36.71 ± 0.36^c^	145.56	46.65 ± 0.28^d^	175.55
**8b**	27.67 ± 0.54^d^	85.09	36.93 ± 0.38^c^	118.14
Control	14.95 ± 0.70^e^		16.93 ± 0.54^b^	

Whereby, means ± SE (standard error) of 3 replicates of 50 of 4th larvae each. Distinct letters designate significant variances between treatments as stated by Duncan’s test. LSD = 0.05 was 1.341 U/L for ALT and was 1.435 U/L for AST. % of control = (test−control)/control × 100

##### E. Acetyl cholinesterase enzyme activity analysis

Compound **8d** produced a significant highest reduction in the activity of acetyl cholinesterase activity (A-chE) lower than in the control, it was − 65.50%, followed by **8a** (− 49.61%) and **4b** (− 47.24%) lower than in the control test (Table 7). Whereas, the lowest decrease in A-chE activity was induced by **8b** (− 29.96%) compared to the control test.

**Table 7:** A-chE activity of A-chE in Hemolymph of the 4th Instar Larvae of *S. littoralis* later 5 days of treatment with LC_50_ of **8d**, **8a**, **4b**, and **8b** tested compounds

Tested compounds	Acetyl cholinesterase activity (mU/mL)	% of control
**8d**	264.11 ± 0.76^e^	− 65.50
**8a**	385.81 ± 0.86^d^	− 49.61
**4b**	403.90 ± 1.06^c^	− 47.24
**8b**	536.19 ± 1.30^b^	− 29.96
Control	765.53 ± 1.63^a^	

Whereby, means ± SE (standard error) of 3 replicates of 50 of 4th larvae each. Distinct letters designate significant variances between treatments as stated by Duncan’s test. LSD = 0.05 was 3.663 mU/mL for acetyl cholinesterase. % of control = (test−control)/control × 100

## Theoretical approaches

**Fig. 2:**
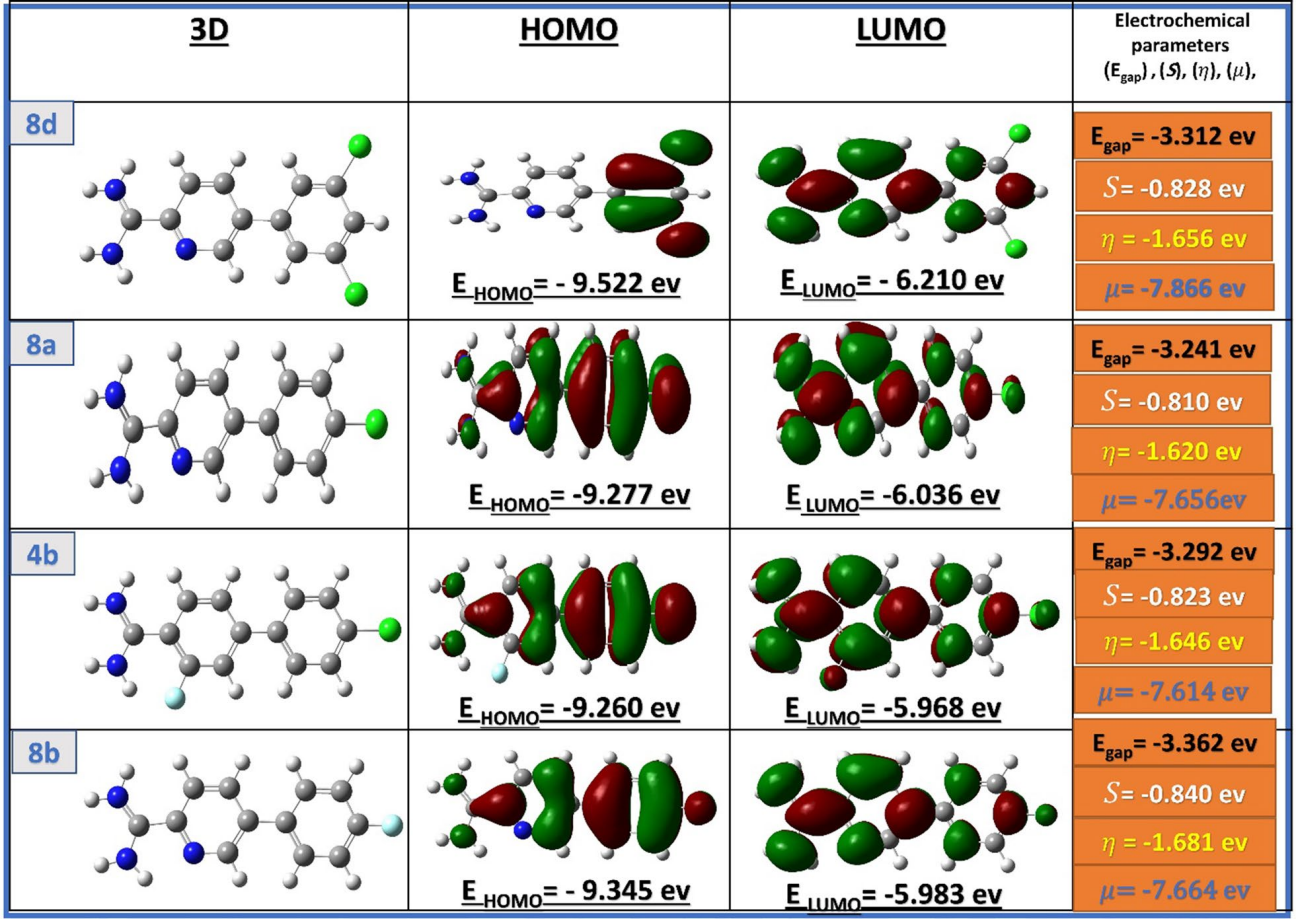
The optimized geometrical structures, spatial distribution of calculated HOMO, LUMO orbitals, and global chemical reactivity descriptors of highly potential tested compounds **8d**, **8a**, **4b** and **8b**

Computational calculations were performed using Gaussian 09 program, in the gaseous state, the DFT approach B3LYP as function and 6-311G (d,p) as basis set to investigate chemical reactivity and stability of the geometry optimization preliminary results of expected biological evaluation of the synthesized compounds via studying of different electrochemical parameters such as total energies, spatial distribution of HOMO and LUMO orbitals, energy gap, hardness (η) and global softness (*S*), which are depicted in Fig. 2, were carried out through using consequent Eqs. (1, 2, 3, 4) [32–35].

1 $$\Delta E=\left({E}_{HOMO}-{E}_{LUMO}\right)$$

2 $$\eta = \frac{1}{2}\left({E}_{HOMO}-{E}_{LUMO}\right)$$

3 $$S=\frac{1}{2}\eta$$

4 $$\mu = \frac{1}{2}\left({E}_{HOMO}+{E}_{LUMO}\right)$$

The obtained results showed that highly active compounds in order **8d**, **8a**, **4b**, and **8b** having high softness values than other tested compounds. As global chemical reactivity descriptors such as the energy gap, softness, and chemical potential values, are critical theoretical tools for clarifying the chemical reactivity of the synthesized compounds and the biological activity is directly proportional to increasing with their values. Whereby, a molecule that has a high softness and chemical potential values, is called a soft molecule. Soft molecules are more reactive than hard molecules as they have the ability to donate electrons more easily to acceptors than other molecules. Besides, biological activity depends mainly on the ability of the molecule to offer electrons easily to the acceptor. As seen in the highly potent compounds **8d**, **8a**, **4b**, and **8b**, there are slight differences in their band gaps, softness, and chemical potential values (Fig. 2). [36, 37]. Additionally, the tested molecules display noticeable chemical reactivity descriptors that facilitate prolonged interactions with the enzymes of cotton leafworms. Subsequently, robust disorders in their biochemical and metabolic processes will be carried out, leading to decrease leafworms, growth rates or reduce in their productivity or cause the death of the targeted worms collectively, according to the above-mentioned results.

## Structure activity relationship (SAR)

The main reason for the higher potency of compound **8d** than other test molecules might be rationalized due to the presence of pyridine, amidine moieties, and di-substitution of chloro-atoms enhanced the insecticidal activity [38]. Whereas, the other compounds **8a**, and **8b** containing only one halogenated atom. Whereas, there has been a rise in the number of commercial products containing ‘mixed’ different halogens [28]; in order to explore the essential moiety in insecticidal activity of the synthesized series and aim to investigate the insecticidal activity of molecules that has a mixed or different halogenated atoms chlorine and fluorine atoms we synthesized parent disubstituted halogenated biphenyl compound **4b**. However, compound **4b** showed lower insecticidal potency than compounds **8d** and **8a**; and the addition of different halogens did not impart any benefits; this postulate proves that the most essential active moieties in the synthesized compounds are pyridine and amidine moieties.

## Experimental

### Chemistry

All devices, equipment, materials, and methods are shown and discussed in detail in the ESI.

#### General methodology of Suzuki coupling methodology for the preparation of biphenyl carbonitriles 3a-d was carried out according to the described method [39]

*4*^*ʹ*^*-Chloro-[1,1*^*ʹ*^*-biphenyl]-4-carbonitrile (****3a****)* A pale-yellow solid; yield = 69%, m.p. = 126.5–128 °C, Lit. [40] m.p. = 125–127 ℃, Lit. [41] m.p. = 129–130 ℃; R_f_ = 0.81, EtOAc/petroleum ether (60–80 °C) (1:4). IR (KBr, ν^ʹ^/cm^−1^): 3067, 3035 (sp^2^ C–H), 2225 (CN), 1604, 1522, 1481 (C=C). ^1^H-NMR; *δ* 7.54 (d, *J* = 9 *Hz*, 2H, Ar–H of *para*-chlorophenyl), 7.75 (d, *J* = 9 *Hz*, 2H, Ar–H of benzonitrile), 7.86 (d, *J* = 9 *Hz*, 2H, Ar–H of benzonitrile), 7.91 ppm (d, *J* = 9 *Hz,* 2H, Ar–H of *para*-chlorophenyl). MS (EI) (m/e, %) for C_13_H_8_ClN; 213.77, 215.63 (M^+^, 51.71, 16.19%; chlorine isotopes), 120.09 (Base peak, 100%).

*4*^*ʹ*^*-Chloro-3-fluoro-[1,1*^*ʹ*^*-biphenyl]-4-carbonitrile (****3b****)* An off-white solid; yield = 70%; m.p. = 174–175 °C, Lit. [42] m.p. = 175–177 ℃; R_f_ = 0.88, EtOAc/petroleum ether (60–80 °C) (1:4). IR (KBr, ν^ʹ^/cm^−1^): 3086 (sp^2^ C–H), 2231 (CN), 1618, 1554, 1482 (C=C). MS (EI) (m/e, %) for C_13_H_7_ClFN; 231.15, 233.15 (M^+^, 80.32, 27.18%; chlorine isotopes), 75 (Base peak, 100%).

*3,4*^*ʹ*^*-Difluoro-[1,1*^*ʹ*^*-biphenyl]-4-carbonitrile (3c)* A white solid; yield = 73%; m.p. = 118–119 °C. Lit. [43]; R_f_ = 0.91, EtOAc/petroleum ether (60–80 °C) (1:4). IR (KBr, ν^ʹ^/cm^−1^): 3096, 3069 (sp^2^ C–H), 2231 (CN), 1617, 1596, 1560 (C=C). ^1^H-NMR; *δ* 7.33–7.36 (m, 2H, Ar–H of *para*-fluorophenyl), 7.73 (d, *J* = 8.0 *Hz*, 1H, Ar–H of benzonitrile), 7.85–7.90 ppm (m, 3H), 7.97–8.00 (m, 1H, Ar–H of benzonitrile). MS (EI) (m/e, %) for C_13_H_7_F_2_N; 215.15 (M^+^, Base peak, 100%).

*3-Fluoro-3*^*ʹ*^*,5*^*ʹ*^*-dimethoxy-[1,1*^*ʹ*^*-biphenyl]-4-carbonitrile (****3d****)* A pale-yellow solid yield; 82%, m.p. = 114–115 °C; R_f_ = 0.82, EtOAc/petroleum ether (60–80 °C) (1:4). IR (KBr, ν^ʹ^/cm^−1^): 2953, 2847 (sp^3^ C–H), 2230 (CN), 1602, 1561(C=C). ^1^H-NMR; *δ* 3.81 (s, 6H, *meta*-dimethoxy-H’s), 6.59 (s, 1H, Ar–H of dimethoxyphenyl), 6.91 (s, 2H, Ar–H of dimethoxyphenyl), 7.76 (dd, *J* = 8, 1.5 *Hz*, 1H, Ar–H of fluorobenzonitrile), 7.92 (d, *J* = 1.5 *Hz*, 1H, Ar–H of fluorobenzonitrile), 7.96–7.99 ppm (m, 1H, Ar–H of fluorobenzonitrile). MS (EI) (m/e, %) for C_15_H_12_FNO_2_; 257.25 (M^+^, Base peak, 100%).

#### Preparation methodology for biphenylcarboxamidine derivatives 4a–d

Biphenylcarbonitrile derivatives **3a**–**d** (1.5 mmol) were allowed to react with LiN(TMS)_2_ (1M solution in THF, 8 mL, 8 mmol) at room temperature with stirring overnight. After that, ethanol-HCl (gas) solution (12 mL, 1.25 M) was added and the reaction mixture was stirred for another 6 h. The resulting mixture was diluted with diethyl ether (Et_2_O) and the formed solid was filtered off. Then, the resultant biphenylcarboxamidine derivative was neutralized with NaOH (1N) and the formed monoamidine-free base was collected, washed with water. Finally, the target biphenylcarboxamidines hydrochloride salts **4a**–**d** were made from their corresponding free bases on treatment with ethanolic-HCl(gas) solution for overnight, the solid formed was filtered off after addition of Et_2_O.

*4*^*ʹ*^*-Chloro-[1,1*^*ʹ*^*-biphenyl]-4-carboxamidine hydrochloride salt (****4a****)* An off white solid; yield = 74%; m.p. = 282–284 °C. IR (KBr, ν^ʹ^/cm^−1^): 3232 (NH), 3057 (sp^2^ C–H), 1720, 1670, 1608, 1540 (C=N & C=C). ^1^H-NMR; *δ* 7.56 (d, *J* = 6.5 *Hz*, 2H, Ar–H of *para*-chlorophenyl), 7.81 (d, *J* = 6.5 *Hz*, 2H, Ar–H of *para*-chlorophenyl), 7.92–7.96 (m, 4H, Ar–H of benzamidine), 9.31 (s, 2H, NH_2_ exchangeable with D_2_O), 9.52 ppm (s, 2H, ^+^NH_2_ exchangeable with D_2_O). ^13^C-NMR; *δ* 127.01 (1C), 127.05 (2C), 128.94 (4C), 129.18 (2C), 133.69 (1C), 137.17 (1C), 143.85 (1C), 165.18 (1C). MS (EI) (m/e, %) for C_13_H_11_ClN_2_; 230.82, 232.46 (M^+^, 44.03, 9.56%; chlorine isotopes), 219.17 (Base peak, 100%).

*4-Chloro-3-fluoro-[1,1-biphenyl]-4-carboxamidine hydrochloride salt (****4b****)* An off white solid; yield = 77%; m.p. = 284–286 °C. IR (KBr, ν^ʹ^/cm^−1^): 3166 (NH), 1673, 1623, 1540 (C=N & C=C). ^1^H-NMR; *δ* 7.58 (d, *J* = 8.0 *Hz*, 2H, Ar–H of *para*-chlorophenyl), 7.75–7.80 (m, 2H, Ar–H of fluorobenzamidine), 7.83–7.88 (m, 3H, Ar–H), 9.51 (s, 2H, NH_2_ exchangeable with D_2_O), 9.56 ppm (s, 2H, ^+^NH_2_ exchangeable with D_2_O). ^19^F-NMR (DMSO-*d*_*6*_); − *δ* 112.57 ppm (using TFA as external standard). MS (EI) (m/e, %) for C_13_H_10_ClFN_2_; 248.76 (M^+^, 14.95%), 250.35 (M^+^ + 2, 8.23%), 43.22 (Base peak, 100%).

*3,4*^*ʹ*^*-Difluoro-[1,1*^*ʹ*^*-biphenyl]-4-carboxamidine hydrochloride salt (****4c****)* An off white solid; yield = 74%; m.p. = 282–284 °C. IR (KBr, ν^ʹ^/cm^−1^): 3250 (NH), 3060 (sp^2^ C–H), 1680, 1629, 1546 (C=N & C=C). ^1^H-NMR; *δ* 7.33–7.38 (m, 2H, Ar–H of *para-*fluorophenyl), 7.73–7.77 (m, 2H, Ar–H of *para*-fluorophenyl), 7.83–7.88 (m, 3H, Ar–H of fluorobenzamidine), 9.49 (s, 2H, NH_2_ exchangeable with D_2_O), 9.55 ppm (s, 2H, ^+^NH_2_ exchangeable with D_2_O). MS (EI) (m/e, %) for C_13_H_10_F_2_N_2_; 232.99 (M^+^, 84.42%), 119.53 (Base peak, 100%).

*3-Fluoro-3*^*ʹ*^*,5*^*ʹ*^*-dimethoxy-[1,1*^*ʹ*^*-biphenyl]-4-carboxamidine hydrochloride salt (****4d****)* An off white solid; yield = 79%; m.p. = 226–228°C. IR (KBr, ν^ʹ^/cm^−1^): 3381, 3245 (2NH_2_), 3001 (sp^2^ C–H), 2841 (sp^3^ C–H), 1675, 1598, 1460 (C=N & C=C). ^1^H-NMR; *δ* 3.81 (s, 6H, *meta*-dimethoxy-H’s), 6.59 (s, 1H, Ar–H of dimethoxyphenyl), 6.91 (s, 2H, Ar–H of dimethoxyphenyl), 7.73–7.78 (m, 2H, Ar–H of fluorobenzamidine), 7.85–7.88 (d, 1H, *J* = 10.5 Ar–H of fluorobenzamidine), 9.49 (s, 2H, NH_2_ exchangeable with D_2_O), 9.55 ppm (s, 2H, ^+^NH_2_ exchangeable with D_2_O). MS (EI) (m/e, %) C_15_H_15_FN_2_O_2_; 274.95 (M^+^, 47.95), 167.89 (Base peak, 100%).

#### General methodology for the preparation of picolinonitrile derivatives 7a–d

Picolinonitrile derivatives **7a**–**d** were prepared to adopt the same Suzuki coupling conditions used for the synthesis of biphenyl carbonitriles **3a**–**d**, starting with 5-bromopicolinonitrile and the appropriate phenylboronic acid.

*5-(4-Chlorophenyl)picolinonitrile (****7a****)* A yellow solid; yield = 66%; m.p. = 144–145 °C; R_f_ = 0.84, EtOAc/petroleum ether (60–80 °C) (1:4). IR (KBr, ν^ʹ^/cm^−1^): 3055 (sp^2^ C–H), 2228 (CN), 1653, 1595, 1499 (C=N & C=C). ^1^H-NMR; *δ* 7.61 (d, *J* = 8.4 *Hz,* 2H, Ar–H of *para*-chlorophenyl), 7.88 (d, *J* = 8.4 *Hz*, 2H, Ar–H of *para*-chlorophenyl), 8.13 (d, *J* = 8.1 *Hz*, 1H, Ar–H of picolinonitrile), 8.36 (dd, *J* = 8.1, 2.4 *Hz*, 1H, Ar–H of picolinonitrile), 9.10 ppm (s, 1H, Ar–H of picolinonitrile). ^13^C-NMR; *δ* 117.57 (1C), 129.10 (2C), 129.21 (2C), 129.29 (1C), 131.40 (1C), 134.07 (1C), 134.46 (1C), 135.40 (1C), 137.70 (1C), 149.22 (1C). MS (EI) (m/e, %) for C_12_H_7_ClN_2_; 214.10, 216.15 (M^+^, 85.36, 27.28%; chlorine isotopes), 75.10 (Base peak, 100%).

*5-(4-Fluorophenyl)picolinonitrile (****7b****)* A white solid; yield = 64%; m.p. = 146–147 °C, Lit. [44]; R_f_ = 0.84, EtOAc /petroleum ether (60–80 °C) (1:4). IR (KBr, ν^ʹ^/cm^−1^): 3066 (sp^2^ C–H), 2231 (CN), 1605, 1561, 1515 (C=N& C=C). ^1^H-NMR; *δ* 7.36–7.42 (m, 2H, Ar–H of *para*-fluorophenyl), 7.88–7.92 (m, 2H, Ar–H of *para*-fluorophenyl), 8.12 (d, *J* = 8.1 *Hz*, 1H, Ar–H of picolinonitrile), 8.34 (dd, *J* = 8.1, 1.5 *Hz*, 1H, Ar–H of picolinonitrile), 9.09 ppm (s, 1H, Ar–H of picolinonitrile). MS (EI) (m/e, %) for C_12_H_7_FN_2_; 198.10 (M^+^, Base peak, 100%).

*5-(3,5-Dimethoxyphenyl)picolinonitrile *(***7c***) A buff solid; yield = 75%; m.p. = 140–141 °C; R_f_ = 0.72, EtOAc /petroleum ether (60–80 °C) (1:4). IR (KBr) ν^ʹ^/cm^−1^: 2965 (sp^3^ C–H, stretch), 2227 (CN, stretch), 1602, 1460 (C=N & C=C, stretch). ^1^H-NMR; *δ* 3.83 (s, 6H, *meta*-dimethoxy-H’s), 6.62 (s, 1H, Ar–H of dimethoxyphenyl), 6.95 (s, 2H, Ar–H of dimethoxyphenyl), 8.10 (d, *J* = 7.5 *Hz*, 1H, Ar–H of picolinonitrile), 8.35 (d, *J* = 8.1 *Hz*, 1H, Ar–H of picolinonitrile), 9.10 ppm (s, 1H, Ar–H of picolinonitrile). ^13^C-NMR; *δ* 55.48 (2C), 101.24 (1C), 105.43 (2C), 117.65 (1C), 129.01 (1C), 131.32 (1C), 135.61 (1C), 137.25 (1C), 138.86 (1C), 149.48 (1C), 161.13 (2C). MS (EI) (m/e, %) for C_14_H_12_N_2_O_2_; 240.20 (M^+^, Base peak, 100%).

*5-(3,5-Dichlorophenyl)picolinonitrile (****7d****)* A white solid; yield = 77%; m.p. = 158–160 °C; R_f_ = 0.87, EtOAc/petroleum ether (60–80 °C) (1:4). IR (KBr, ν^ʹ^/cm^−1^): 3078 (sp^2^ C–H), 2239 (CN), 1571, 1478, 1426 (C=N & C=C). ^1^H-NMR; *δ* 7.74 (s, 1H, *meta*-dichlorophenyl-H), 7.95 (s, 2H, *meta*-dichlorophenyl-H’s), 8.15 (d, *J* = 8.1 *Hz*, 1H, Ar–H of picolinonitrile), 8.45 (dd, *J* = 8.1, 2.4 *Hz*, 1H, Ar–H of picolinonitrile), 9.16 ppm (s, 1H, Ar–H of picolinonitrile). MS (EI) (m/e, %) for C_12_H_6_Cl_2_N_2_; 248.10 (M^+^, 59%), 249.05 (12.66%), 251.10 (5.69%): two chlorine isotopes), 99.15 (Base peak, 100%).

#### General methodology for preparation of picolinamidine derivatives 8a–d

The picolinamidines **8a**–**d** were prepared using the same methodology used for formation of monoamidines **4a**–**d**, starting with picolinonitriles **7a**–**d**.

*5-(4-Chlorophenyl)picolinamidine hydrochloride salt (****8a****)* A buff solid; yield = 61%; m.p. = 232–234 °C. IR (KBr, ν^ʹ^/cm^−1^): 3380, 3272 (2NH_2_), 3061 (sp^2^ C–H), 1688, 1644, 1588 (C=N & C=C). ^1^H-NMR; *δ* 7.62 (d, *J* = 9 *Hz*, 2H, Ar–H of *para*-chlorophenyl), 7.92 (d, *J* = 9 *Hz*, 2H, Ar–H of *para*-chlorophenyl), 8.44 (d, *J* = 9 *Hz*, 1H, Ar–H of picolinamidine), 8.50 (d, *J* = 2.5 *Hz*, 1H, Ar–H of picolinamidine), 9.14 (s, 1H, Ar–H of picolinamidine), 9.46 (s, 2H, NH_2_ exchangeable with D_2_O), 9.66 ppm (s, 2H, ^+^NH_2_ exchangeable with D_2_O). ^13^C-NMR; *δ* ppm 123.48 (1C), 129.29 (2C), 129.32 (2C), 134.17 (1C), 134.44 (1C), 135.83 (1C), 138.42 (1C), 142.83 (1C), 147.72 (1C), 161.58 (1C). MS (EI) (m/e, %) for C_12_H_10_ClN_3_; 231.00 (M^+^, 10.95), 63.95 (Base peak, 100%).

*5-(4-Fluorophenyl)picolinamidine hydrochloride salt (****8b****)* A buff solid; yield = 64%; m.p. = 228–230 °C. IR (KBr, ν^ʹ^/cm^−1^): 3411 (NH), 3052 (sp^2^ C–H), 1686, 1596, 1535 (C=N & C=C). ^1^H-NMR; *δ* 7.40 (d, *J* = 8.5 *Hz*, 2H, Ar–H of *ortho*-fluorophenyl), 7.96 (d, *J* = 8.5 *Hz*, 2H, Ar–H of *meta*-fluorophenyl), 8.46–8.47 (m, 2H, Ar–H of picolinamidine), 9.12 (s, 1H, Ar–H of picolinamidine), 9.51 (s, 2H, NH_2_ exchangeable with D_2_O), 9.69 ppm (s, 2H, ^+^NH_2_ exchangeable with D_2_O). ^19^F-NMR (DMSO-*d*_*6*_); − *δ* 112.39 ppm (using TFA as external standard). MS (EI) (m/e, %) for C_12_H_10_FN_3_; 215.05 (M^+^, 24.54%), 199 (M^+^ + 1−NH_3_, Base Peak, 100%).

*5-(3*^*ʹ*^*,5*^*ʹ*^*-Dimethoxyphenyl)picolinamidine hydrochloride salt (****8c****)* A buff solid; yield = 72%; m.p. = 230–232 °C. IR (KBr, ν^\^/cm^−1^): 3445, 3375 (2NH_2_), 3142, 3045 (sp^2^ C–H), 2838 (sp^3^ C–H), 1689, 1599, 1535 (C=N & C=C). ^1^H-NMR; *δ* 3.83 (s, 6H, *meta*-dimethoxy-H’s), 6.63 (s, 1H, Ar–H of dimethoxyphenyl), 6.99 (s, 2H, Ar–H of dimethoxyphenyl), 8.42 (d, *J* = 8 *Hz*, 1H, Ar–H of picolinamidine), 8.48 (dd, *J* = 8, 1.5 *Hz*, 1H, Ar–H of picolinamidine), 9.13 (d, *J* = 1.5 *Hz*, 1H, Ar–H of picolinamidine), 9.45 (s, 2H, NH_2_ exchangeable with D_2_O), 9.65 ppm (s, 2H, ^+^NH_2_ exchangeable with D_2_O). ^13^C-NMR; δ 55.50 (2C), 101.05 (1C), 105.57 (2C), 123.36 (1C), 136.02 (1C), 137.32 (1C), 139.55 (1C), 142.74 (1C), 147.93 (1C), 161.16 (2C), 161.61 (1C). MS (EI) (m/e, %) for C_14_H_15_N_3_O_2_; 257.10 (M^+^, 39.08), 241.05 (M^+^ + 1-NH_3_, Base peak, 100%).

*5-(3*^*ʹ*^*,5*^*ʹ*^*-Dichlorophenyl)picolinamidine hydrochloride salt (****8d****)* A buff solid; yield = 70%; m.p. = 286–288 °C. IR (KBr, ν^ʹ^/cm^−1^): 3354 (2NH_2_), 3053 (sp^2^ C–H), 1690, 1656, 1581 (C=N & C=C). ^1^H-NMR; *δ* 7.76 (s, 1H, Ar–H of 3,5-dichlorophenyl), 8.01 (s, 2H, Ar–H of 3,5-dichloroyphenyl), 8.43 (d, *J* = 8 *Hz*, 1H, Ar–H of picolinamidine), 8.57 (d, *J* = 8 *Hz*, 1H, Ar–H of picolinamidine), 9.20 (s, 1H, Ar–H of picolinamidine), 9.45 (s, 2H, NH_2_ exchangeable with D_2_O), 9.66 ppm (s, 2H, ^+^NH_2_ exchangeable with D_2_O). ^13^C-NMR; *δ* 123.37 (1C), 126.26 (2C), 128.71 (1C), 135.03 (1C), 136.54 (2C), 136.90 (1C), 138.88 (1C), 143.56 (1C), 148.12 (1C), 161.43 (1C). MS (EI) (m/e, %) for C_12_H_9_Cl_2_N_3_; 265.32, 266.94 (M + , 5.50; 2.73; two chlorine isotopes), 56.79 (Base peak, 100%).

### Toxicity bioassays

#### Laboratory bioassay

##### Cotton leaf worm (*S. littoralis*, Family; Lepidoptera)

Insecticidal efficacy of the newly synthesized compounds against the 2nd instar larvae of *S. littoralis* (Boisd.) was carried out under laboratory conditions. Eggs of *S. littoralis* were acquired from Cotton Leafworm Research Department, Plant Protection Research Institute, Agricultural Research Center, Dokki, Giza, Egypt, and incubated under well-ordered conditions of 25 ± 1 °C and 70 ± 5% RH and kept away from any chemical contamination till the time of treatment to get a susceptible and homogenous strain [24, 25].

##### Pesticides

**Fig. 3:**
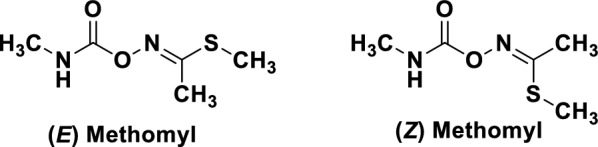
The chemical structures of recommended, methomyl insecticide, lannate 90% SP

(*E*,*Z*)-methyl-*n*-{[(methylamino)carbonyl]oxy}ethanimidothioate which is commercially available under the trade name of lannate 90% soluble powder (SP) (Fig. 3) was purchased from the Central Agricultural Pesticides Laboratory (CAPL) in Dokki, Giza, Egypt.

##### Toxicological studies

The experiments were approved using the leaf dip technique as designated [24, 25]. Six concentrations of each compound (25, 50, 100, 200, 400, and 800 ppm) were formulated as emulsions in DMF, and 0.1% Triton X-100 was used as a surfactant. The emulsions were freshly used after preparation. For larvicidal achievement, fresh castor bean leaves were dipped in the prepared concentrations of each tested compound for 10 s. The treated leaves were left in the shade to dry before being accessible to the larvae. The larvae were allowed to feed on the treated leaves for 48 h and then changed to untreated leaves. Three replicates of 10 larvae each were used for each concentration in addition to the control. Control (check) tests were carried out using the same technique without the tested compound. Larval mortality counts were calculated for 7 days after the exposure period. Mortality was corrected according to Abbott’s formula, and then subjected to probit analysis. The toxicity lines (LC-p lines) were drawn on log concentration–probit paper and statistically analyzed according to Finney’s method, to obtain the LC_50_ and LC_90_ values of different tested compounds so as to determine the utmost operative one. As well, the efficacy of the examined compounds was assessed by comparing the tested compounds with the most effective compound using the following equation [24, 25]:

$$\mathrm{Toxicity \, index }=\frac{{\mathrm{LC}}_{50} \, \mathrm{ of \, the \, utmost \, effective \, compound}}{{\mathrm{LC}}_{50} \, \mathrm{ of \, the \, tested \, compound}}X 100$$

##### Biochemical aspects

Enzymatic activities were assessed in this study using the laboratory strain of 4th instar larvae of *S. littoralis* (Boisd.) after treatment with the tested synthetic biphenyl compounds. At the LC_50_ value of an aqueous emulsion of each insecticide, castor bean leaves were dipped in for 30 s, then left to dry in shade at r.t for 30 min before being offered to the 4th instar larvae of *S. littoralis*. For 48 h the larvae were nurtured on the treated leaves, and then transferred to feed on freshly untreated leaves for 3 days. From approximately 50 larvae, the haemolymph was attained by removing one of the prolegs with forceps; gentle pressure was applied on the larvae with the fingers and extracting the haemolymph with a syringe. The haemolymph was collected in test tubes and stowed in a refrigerator until the determination of the enzyme’s activities [24, 25].

##### Determination of enzyme activities

Alkaline phosphatase (ALK-P) activity was measured according to the reported method [45]. Whereby, acid phosphatase (Acid-p) activity was determined by Fabiny-Byrd &. Ertingshausen. Also, the activity of acetylcholine esterase (AchE) was determined according to the described method. The activities of serum esterases including alanine aminotranferase (ALT) and asparate aminotransferase (AST) enzymes were estimated calorimetrically. Total proteins were estimated by Bradford’s method. Total lipids, lipase, and amylase activities were evaluated according to the reported methods [24, 25].

### Statistical analysis

All biological aspects were analyzed using one-way ANOVA by SPSS 13.0 (SPSS, 2004). Duncan’s Multiple Range Test (DMRT) was used to determine the probability level to compare the differences among some parameter means (P < 0.05) by the Costat system for Windows, Version 6.311, Berkeley, CA, USA, Costat program [46].

## Conclusion

In the current work, new two series of halogenated biphenyl and azaphenyl derivatives have been synthesized via the Suzuki coupling reaction. The effect of halogenated azaphenyl substituted on the development of agrochemical fields via investigating their insecticidal activity and plant growth regulators has been studied. In addition, biochemical aspects and enzyme activities were studied. Consequently, the results of this study will provide a new avenue direction for the further structural optimization of halogenated phenyl pyridine analogs as insecticidal. In this study, the calculated chemical quantum descriptors showed excellent agreement with the experimental data displaying that compounds **8d**, **8a,** and **4b** as promising potential candidates against the cotton leafworm, *S. littoralis*.

### Supplementary Information

**Additional file 1.** **Figure S1:** IR spectrum of compound 3a.** Figure S2:** ^1^H-NMR spectrum of compound 3a. **Figure S3**: Mass spectrum of compound 3a. **Figure S4:** IR Spectrum of compound 3b. **Figure S5:** Mass spectrum of compound 3b. **Figure S6:** ^1^H-NMR spectrum of compound 3c. **Figure S7:** Mass spectrum of compound 3c. **Figure S8: ** IR spectrum of compound 3d. **Figure S9:** ^1^H-NMR spectrum of compound 3d. **Figure S10:** Mass spectrum of compound 3d. **Figure S11:** IR spectrum of compound 4a. **Figure S12:** ^1^H-NMR spectrum of compound 4a. **Figure S13:** ^1^H-NMR (D_2_O) spectrum of compound 4a. **Figure S14:** ^13^C-NMR spectrum of compound 4a. **Figure S15:** Mass spectrum of compound 4a. **Figure S16:** IR spectrum of compound 4b. **Figure S17:** ^1^H-NMR spectrum of compound 4b. **Figure S18:** ^1^H-NMR (D_2_O) spectrum of compound 4b. **Figure S19:** ^19^F-NMR spectrum of compound 4b. **Figure S20:** Mass spectrum of compound 4b. **Figure S21:** IR spectrum of compound 4c. **Figure S22:** ^1^H-NMR spectrum of compound 4c. **Figure S23:** ^1^H-NMR (D_2_O) spectrum of compound 4c. **Figure S24:** Mass spectrum of compound 4c. **Figure S25:** IR spectrum of compound 4d. **Figure S26:** ^1^H-NMR spectrum of compound 4d. **Figure S27:** ^1^H-NMR (D_2_O) spectrum of compound 4d. **Figure S28:** Mass spectrum of compound 4d. **Figure S29:** IR spectrum of compound 7a. **Figure S30:** ^1^H-NMR spectrum of compound 7a. **Figure S31:** ^13^C-NMR spectrum of compound 7a. **Figure S32:** Mass spectrum of compound 7a. **Figure S33:** IR spectrum of compound 7b. **Figure S34:** ^1^H-NMR spectrum of compound 7b. **Figure S35:** Mass spectrum of compound 7b. **Figure S36:** IR spectrum of compound 7c. **Figure S37:** ^1^H-NMR spectrum of compound 7c. **Figure S38:** ^13^C-NMR spectrum of compound 7c. **Figure S39:** Mass spectrum of compound 7c. **Figure S40:** IR spectrum of compound 7d. **Figure S41:** ^1^H-NMR spectrum of compound 7d. **Figure S42:** Mass spectrum of compound 7d. **Figure S43:** IR spectrum of compound 8a. **Figure S44:** ^1^H-NMR spectrum of compound 8a. **Figure S45:** ^1^H-NMR (D_2_O) spectrum of compound 8a. **Figure S46:** ^13^C-NMR spectrum of compound 8a. **Figure S47:** Mass spectrum of compound 8a. **Figure S48:** IR spectrum compound 8b. **Figure S49:** ^1^H-NMR spectrum of compound 8b. **Figure S50:** ^1^H-NMR (D_2_O) spectrum of compound 8b. **Figure S51:** ^19^F-NMR spectrum of compound 8b. **Figure S52:** Mass spectrum of compound 8b. **Figure S53:** IR spectrum of compound 8c. **Figure S54:**^1^H-NMR spectrum of compound 8c. **Figure S55:** ^1^H-NMR spectrum of (D_2_O) compound 8c. **Figure S56:** ^13^C-NMR spectrum of compound 8c. **Figure S57:** Mass spectrum of compound 8c. **Figure S58:** IR spectrum of compound 8d. **Figure S59:** ^1^H-NMR spectrum of compound 8d. **Figure S60:** ^1^H-NMR (D_2_O) spectrum of compound 8d. **Figure S61:** The ^13^C-NMR spectrum of compound 8d. **Figure S62:** Mass spectrum of compound 8d.

## Data Availability

All data and analysis during this study are available in this article and its Additional file 1.
