# Supplementary material for: Synthesis of inventive biphenyl and azabiphenyl derivatives as potential insecticidal agents against the cotton leafworm, Spodoptera littoralis
Source: BMC Chem. 2023 Oct 27;17(1):144. doi: 10.1186/s13065-023-01050-w (PMC10612163; doi:10.1186/s13065-023-01050-w)
Supplement: Supplementary file 1 — Additional file 1. Figure S1: IR spectrum of compound 3a. Figure S2: 1H-NMR spectrum of compound 3a. Figure S3: Mass spectrum of compound 3a. Figure S4: IR Spectrum of compound 3b. Figure S5: Mass spectrum of compound 3b. Figure S6: 1H-NMR spectrum of compound 3c. Figure S7: Mass spectrum of compound 3c. Figure S8: IR spectrum of compound 3d. Figure S9: 1H-NMR spectrum of compound 3d. Figure S10: Mass spectrum of compound 3d. Figure S11: IR spectrum of compound 4a. Figure S12: 1H-NMR spectrum of compound 4a. Figure S13: 1H-NMR (D2O) spectrum of compound 4a. Figure S14: 13C-NMR spectrum of compound 4a. Figure S15: Mass spectrum of compound 4a. Figure S16: IR spectrum of compound 4b. Figure S17: 1H-NMR spectrum of compound 4b. Figure S18: 1H-NMR (D2O) spectrum of compound 4b. Figure S19: 19F-NMR spectrum of compound 4b. Figure S20: Mass spectrum of compound 4b. Figure S21: IR spectrum of compound 4c. Figure S22: 1H-NMR spectrum of compound 4c. Figure S23: 1H-NMR (D2O) spectrum of compound 4c. Figure S24: Mass spectrum of compound 4c. Figure S25: IR spectrum of compound 4d. Figure S26: 1H-NMR spectrum of compound 4d. Figure S27: 1H-NMR (D2O) spectrum of compound 4d. Figure S28: Mass spectrum of compound 4d. Figure S29: IR spectrum of compound 7a. Figure S30: 1H-NMR spectrum of compound 7a. Figure S31: 13C-NMR spectrum of compound 7a. Figure S32: Mass spectrum of compound 7a. Figure S33: IR spectrum of compound 7b. Figure S34: 1H-NMR spectrum of compound 7b. Figure S35: Mass spectrum of compound 7b. Figure S36: IR spectrum of compound 7c. Figure S37: 1H-NMR spectrum of compound 7c. Figure S38: 13C-NMR spectrum of compound 7c. Figure S39: Mass spectrum of compound 7c. Figure S40: IR spectrum of compound 7d. Figure S41: 1H-NMR spectrum of compound 7d. Figure S42: Mass spectrum of compound 7d. Figure S43: IR spectrum of compound 8a. Figure S44: 1H-NMR spectrum of compound 8a. Figure S45: 1H-NMR (D2O) spectrum of compound 8a. Figure S46: 13C-NMR spectrum of compound 8a. Fig [file 13065_2023_1050_MOESM1_ESM.docx]

**Synthesis of inventive biphenyl and azabiphenyl derivatives as potential insecticidal agents against the cotton leafworm, *Spodoptera littoralis***.

**Eslam A Ghaith,^a*^ Hajar A. Ali,^a^ Mohamed A. Ismail,^a^ Abd El-Aziz S. Fouda,^a^ and**

**M. Abd El Salam, ^b^**

(a) *Chemistry Department, Faculty of Science, Mansoura University, 35516 Mansoura, Egypt*

*(b) Plant Protection Research Institute, ARC, Dokki, Giza, Egypt*

*Corresponding author:* ***Eslam A Ghaith***

[*abdelghaffar@mans.edu.eg*](mailto:abdelghaffar@mans.edu.eg)

**Additional file**

**
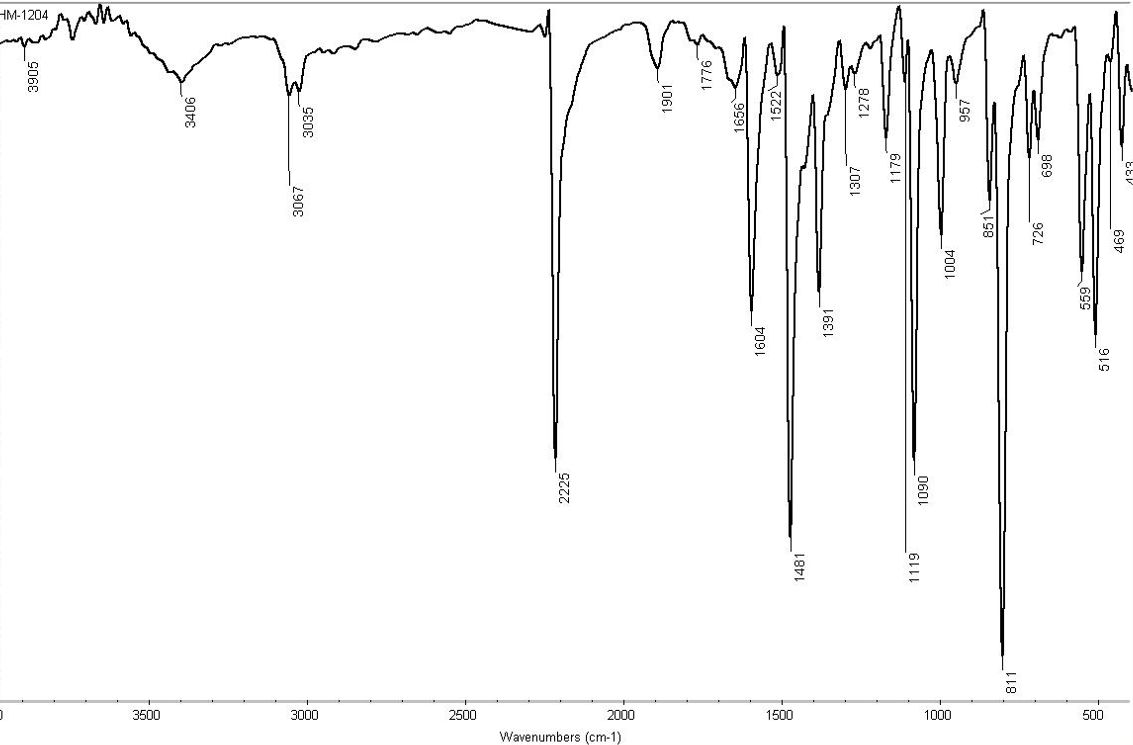
**

**Fig. S1: IR spectrum of Compound 3a**


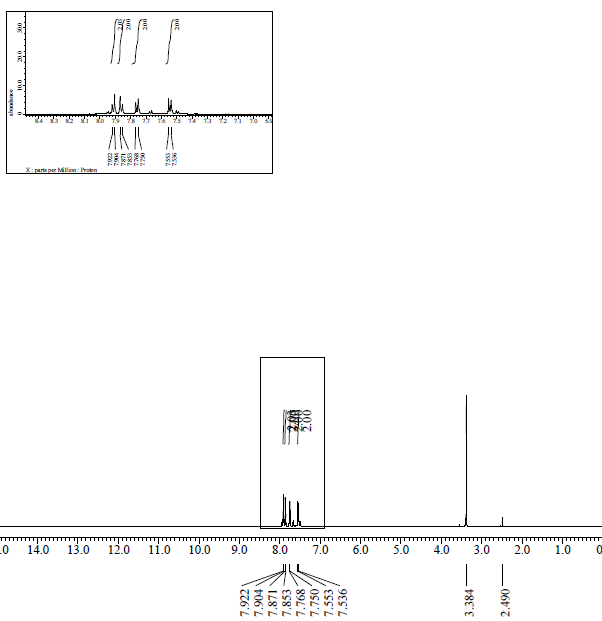

**Fig. S2:  ^1^H-NMR spectrum of compound 3a**


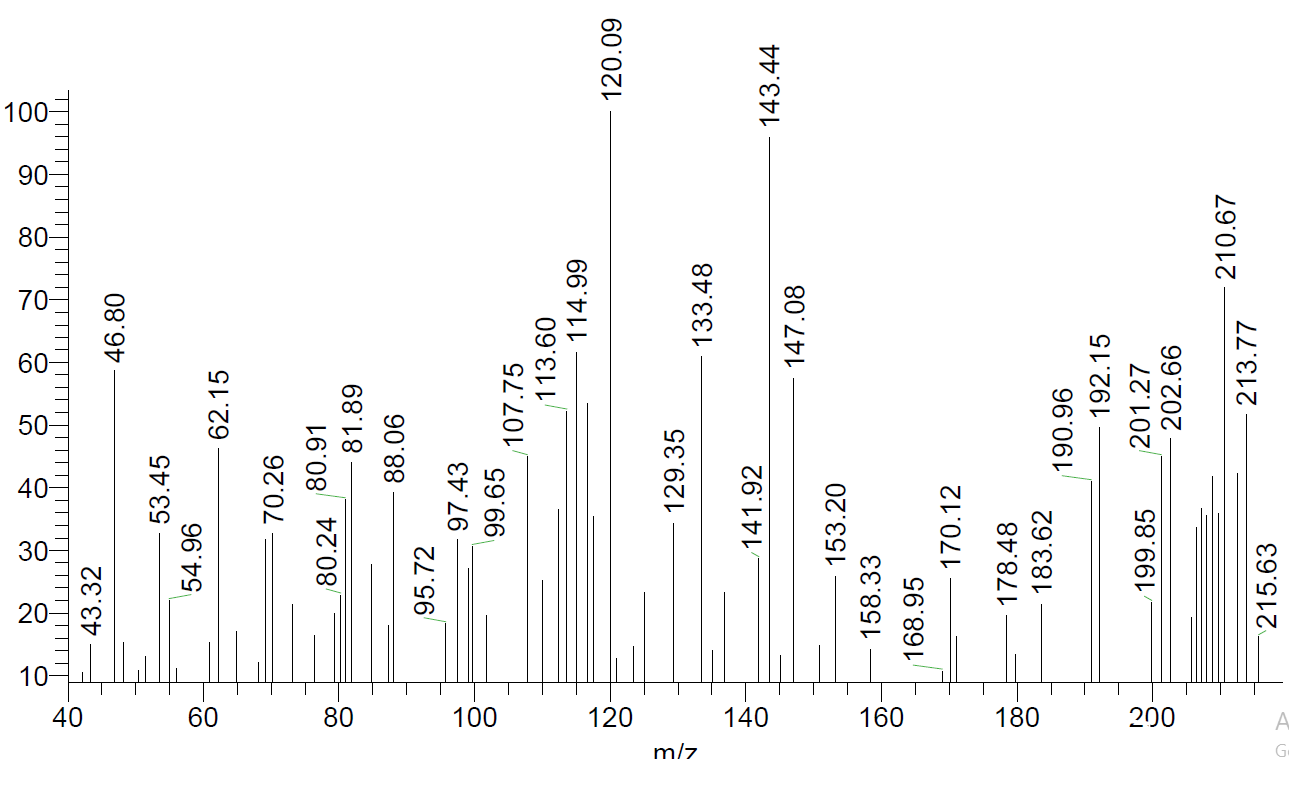

**Fig. S3: Mass spectrum of compound 3a**

**
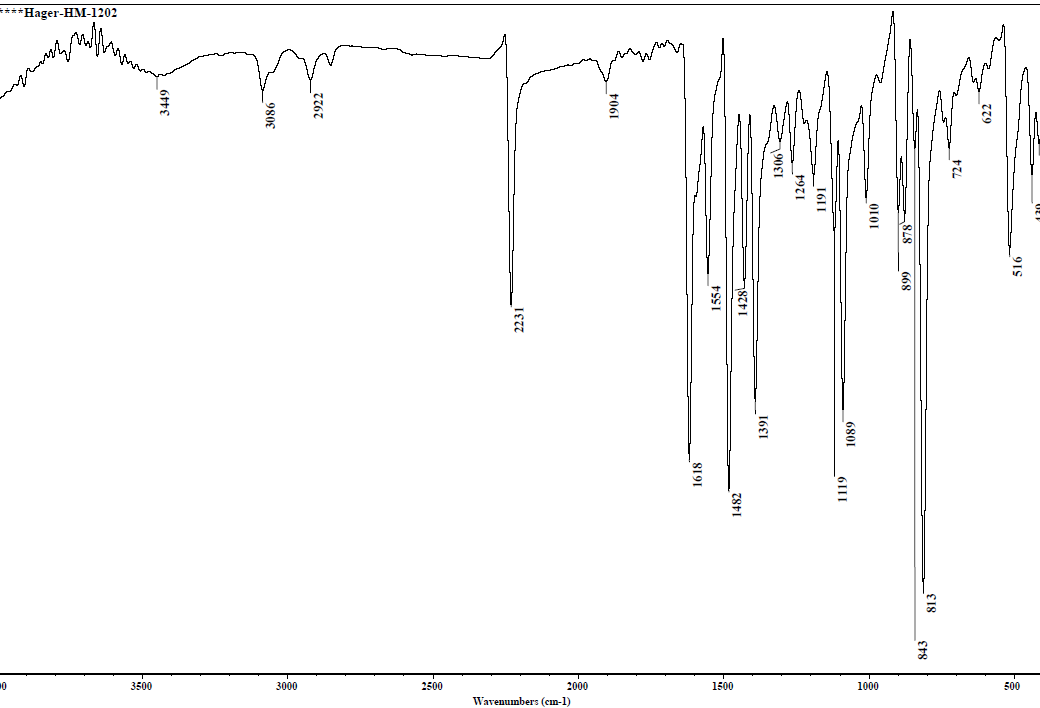
**

**Fig. S4: IR spectrum of compound 3b**


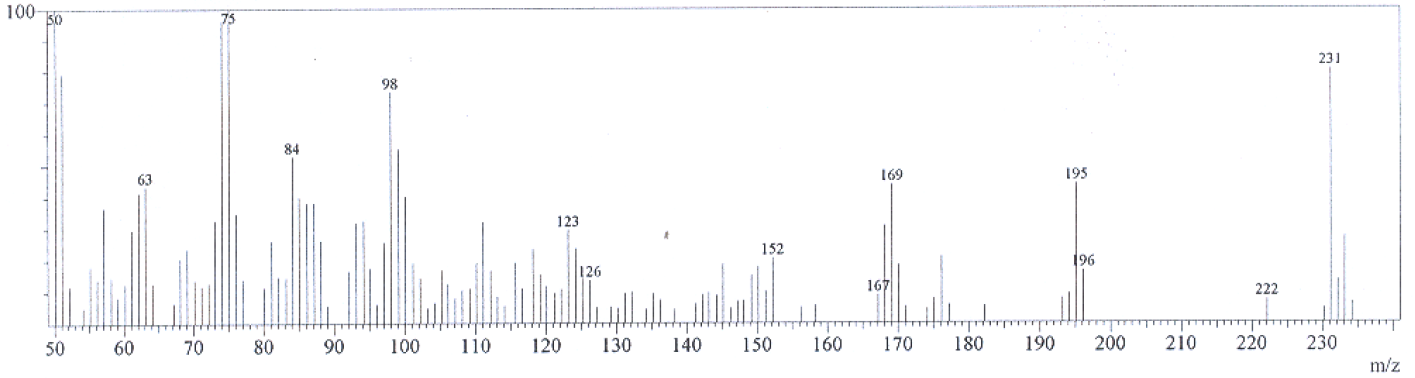

**Fig. S5: Mass spectrum of compound 3b**

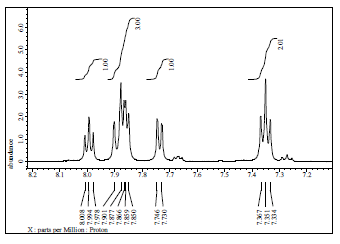


**
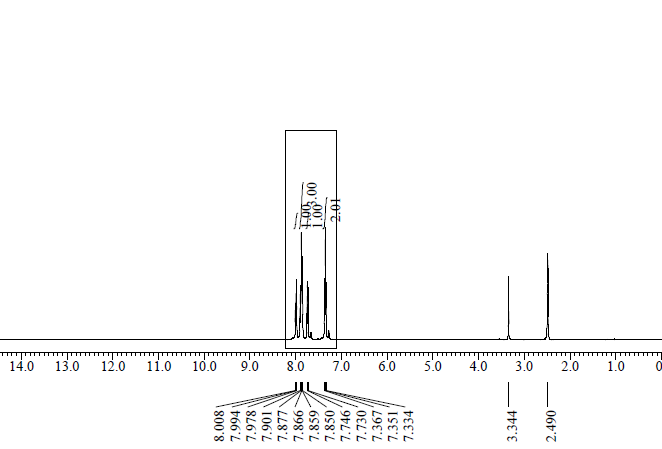
**

**Fig. S6: ^1^H-NMR spectrum of compound 3c**


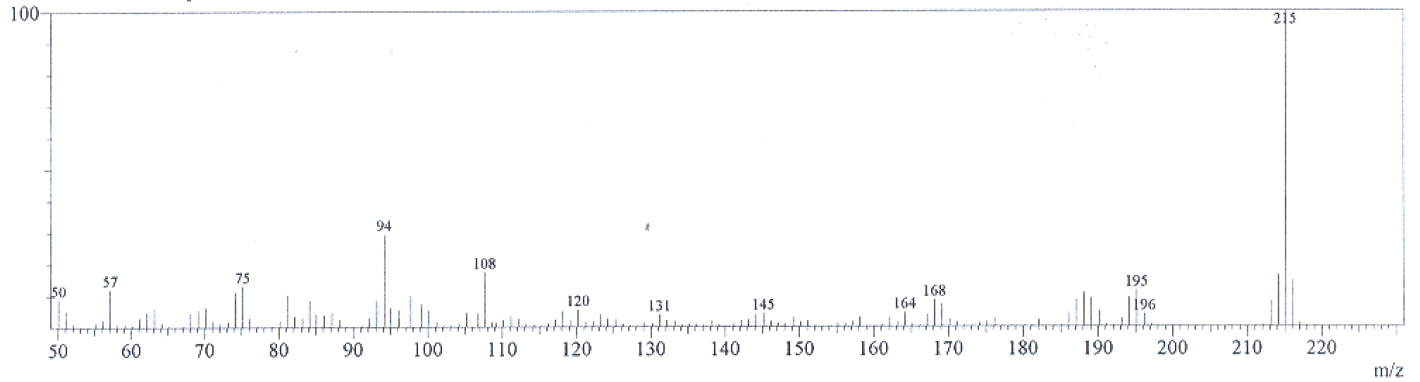

**Fig. S7: Mass spectrum of compound 3c**

**
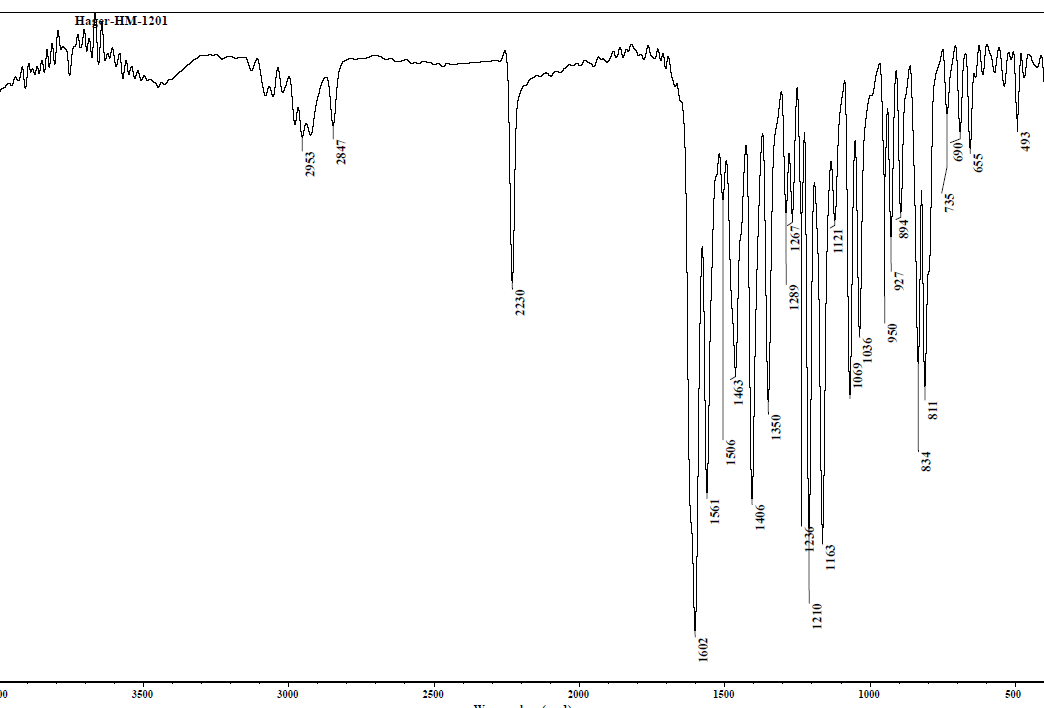
**

**Fig. S8: IR spectrum of compound 3d**

**
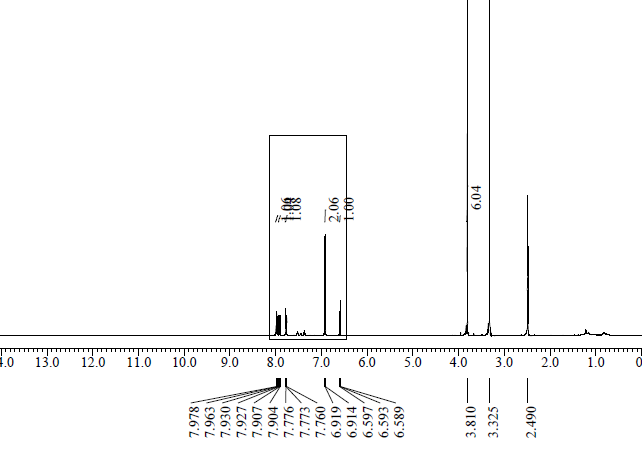
**

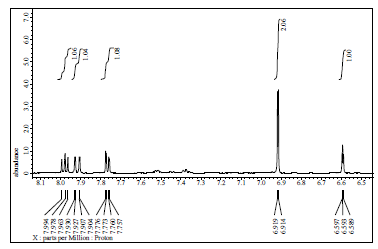


**Fig. S9: ^1^H-NMR spectrum of compound 3d**

**
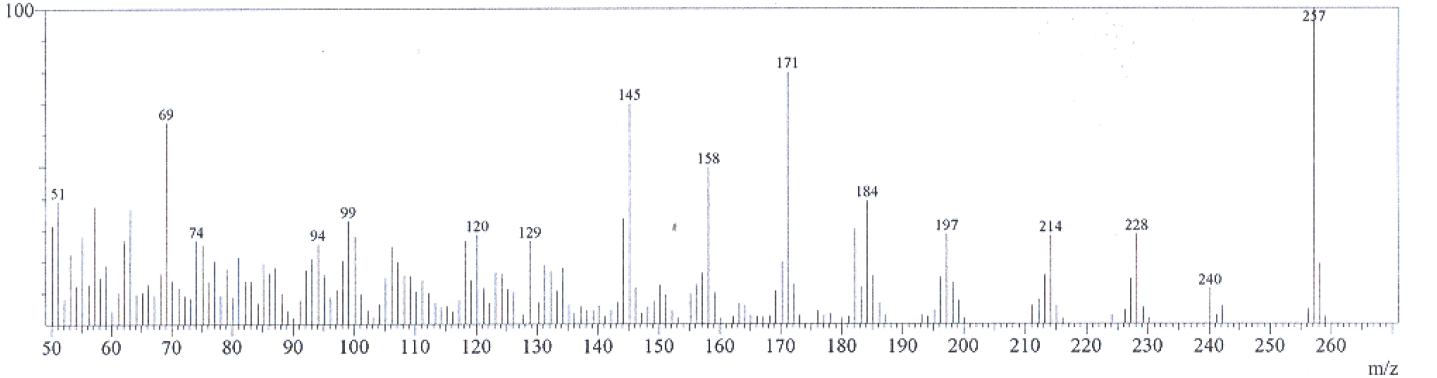
**

**Fig. S10: Mass spectrum of compound 3d**

**
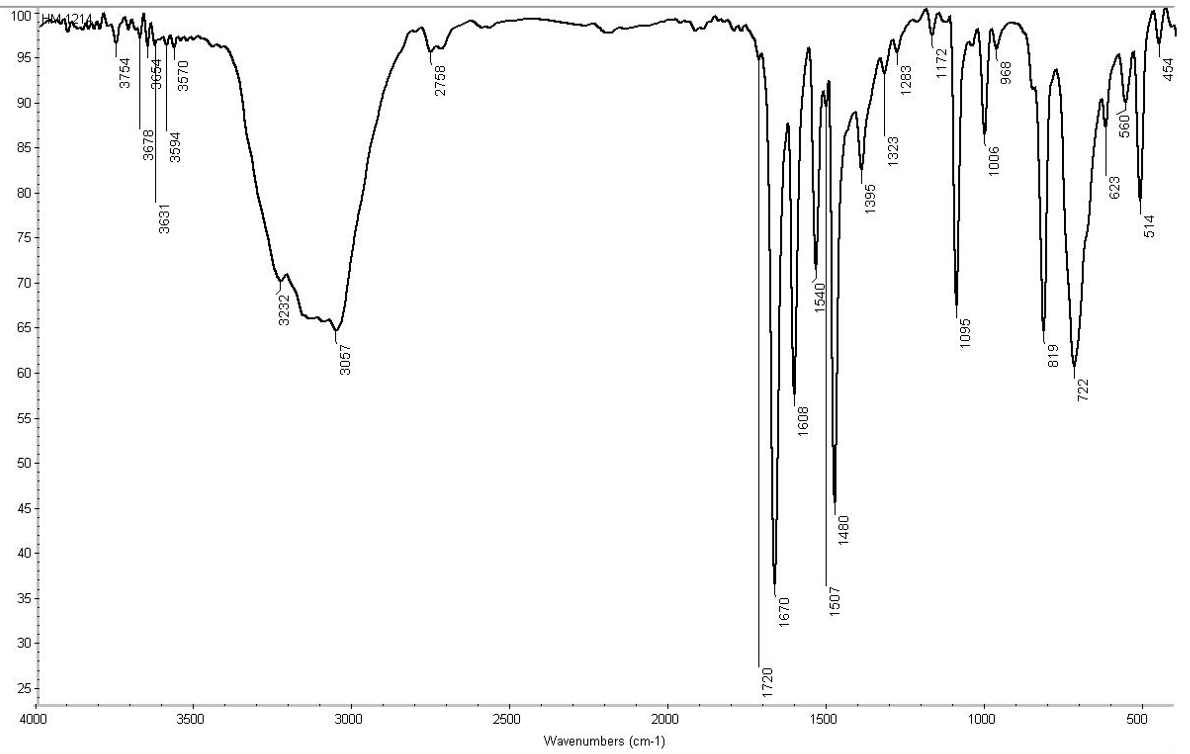
**

**Fig. S11: IR spectrum of compound 4a**


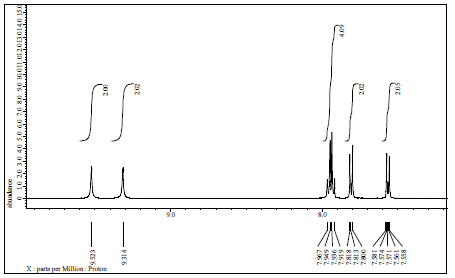
**
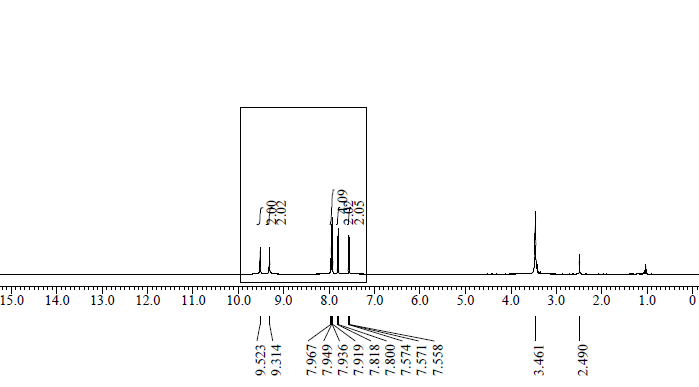
**

**Fig. S12: ^1^H-NMR spectrum of compound 4a**

**
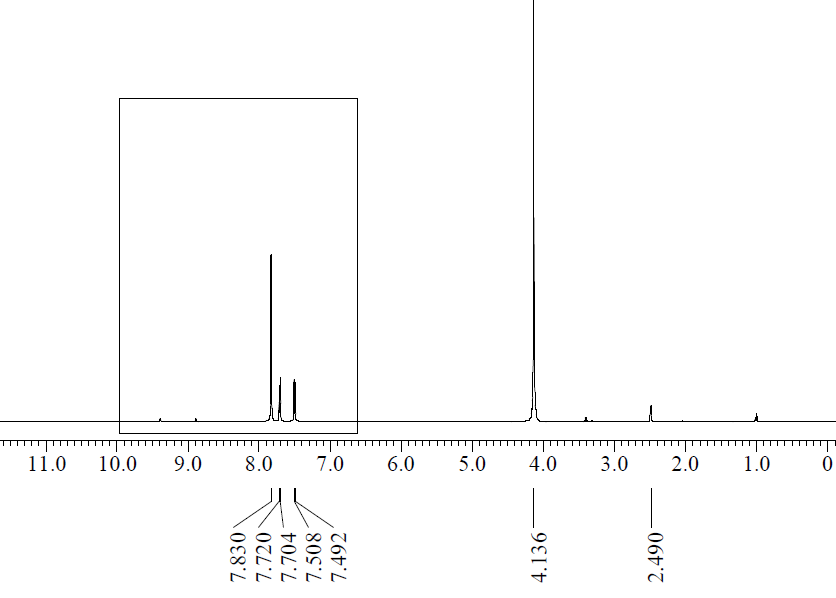
**

**Fig. S13: ^1^H-NMR (D_2_O) spectrum of compound 4a**

**
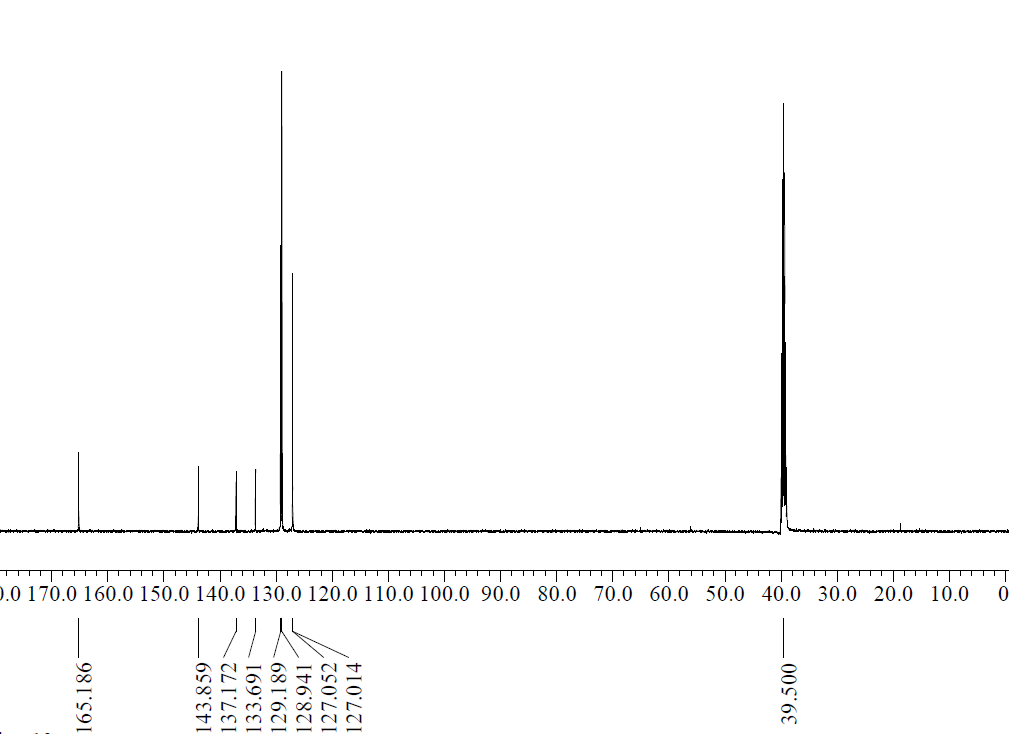
**

**Fig. S14: ^13^C-NMR spectrum of compound 4a**

**
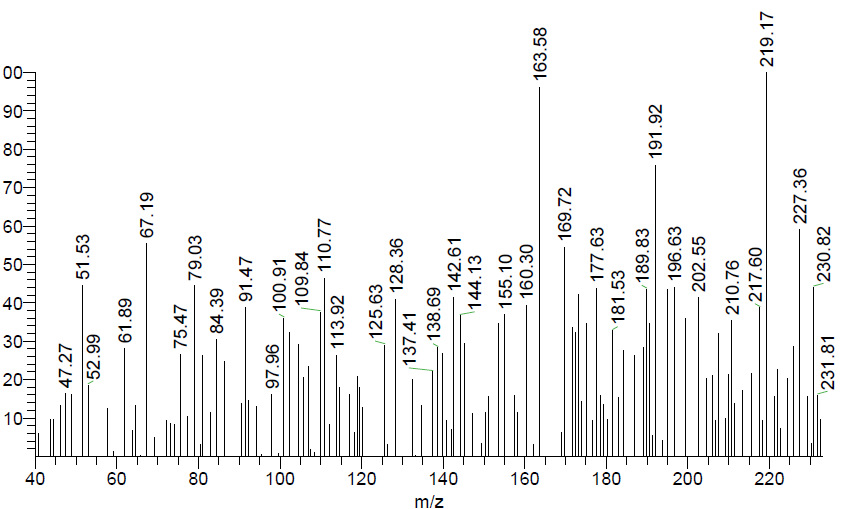
**

**Fig. S15: Mass spectrum of compound 4a**

**
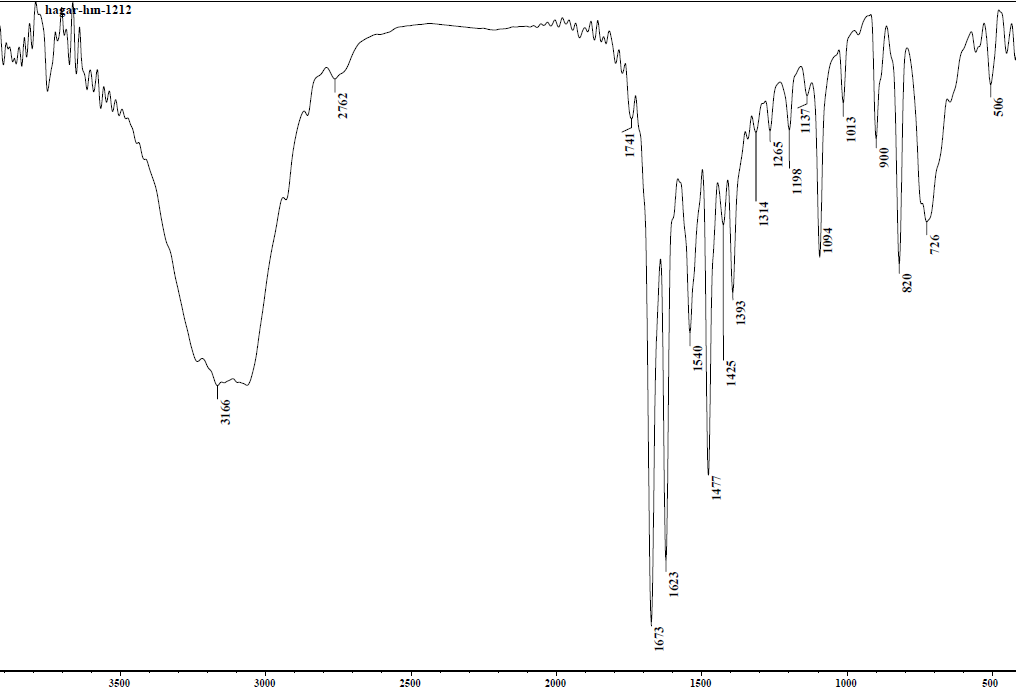
**

**Fig. S16: IR spectrum of compound 4b**


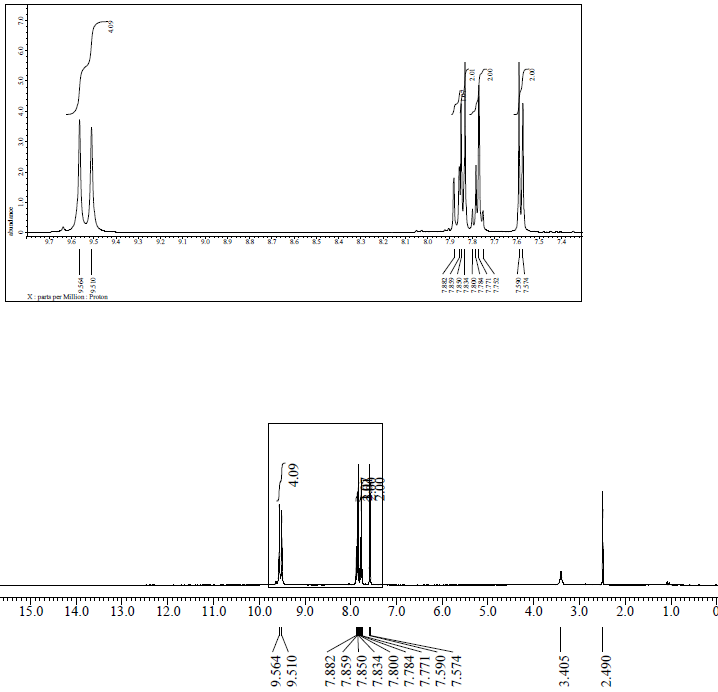

**Fig. S17: ^1^H-NMR spectrum of compound 4b**


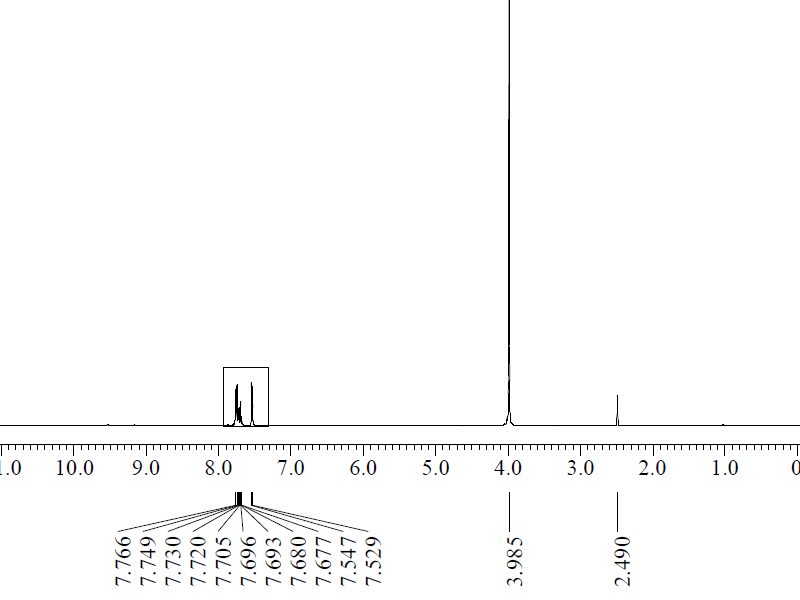

**Fig. S18: ^1^H-NMR (D_2_O) spectrum of compound 4b**

| 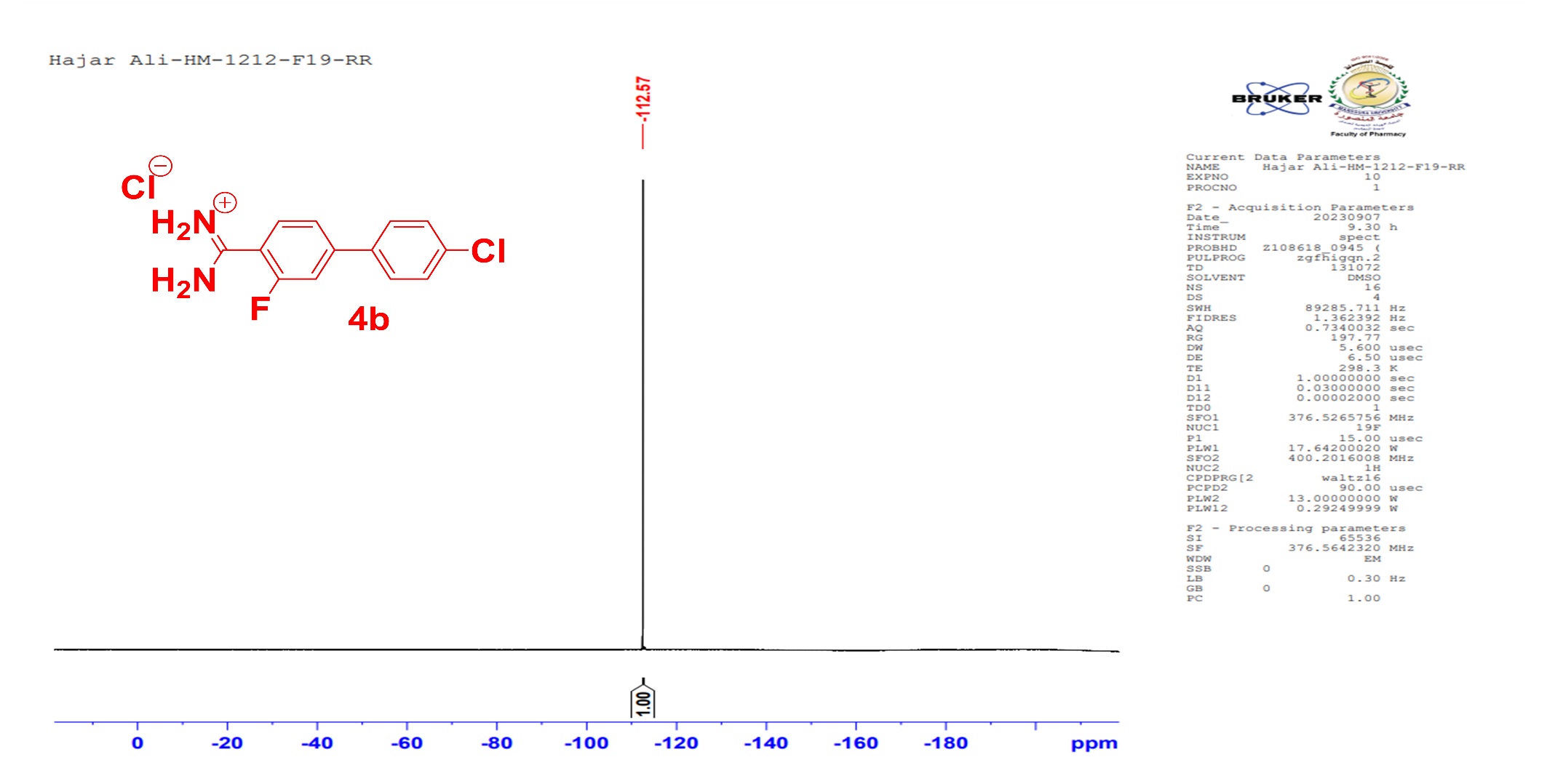 |
| --- |

**Fig. S19: ^19^F-NMR spectrum of compound 4b**


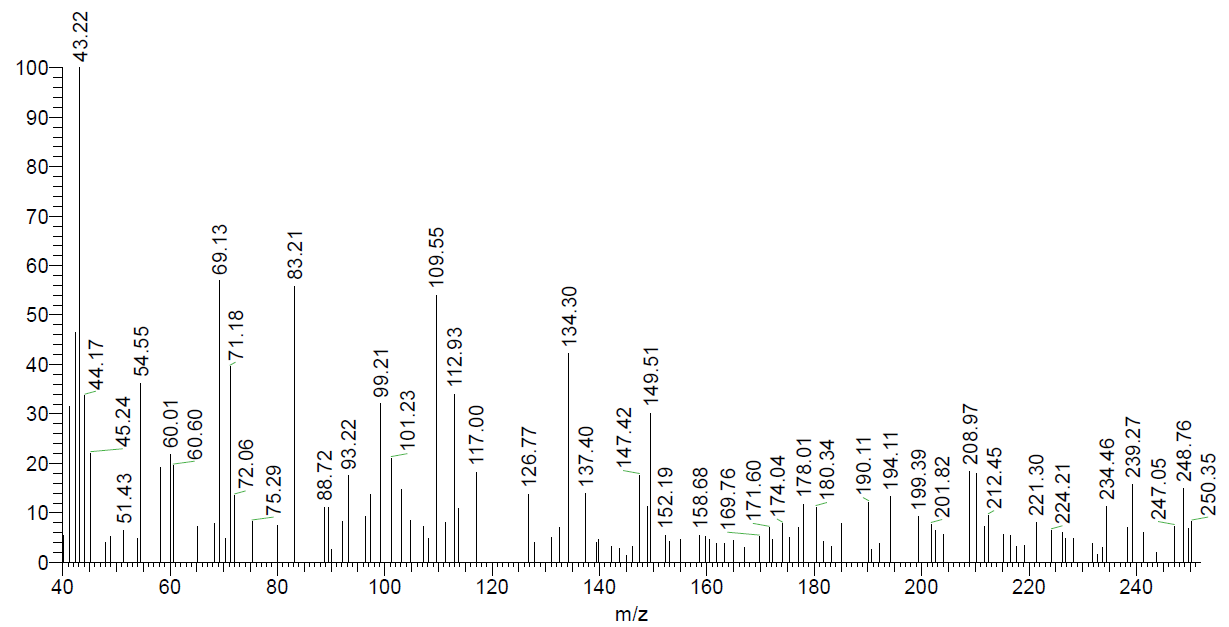

**Fig. S20: Mass spectrum of compound 4b**


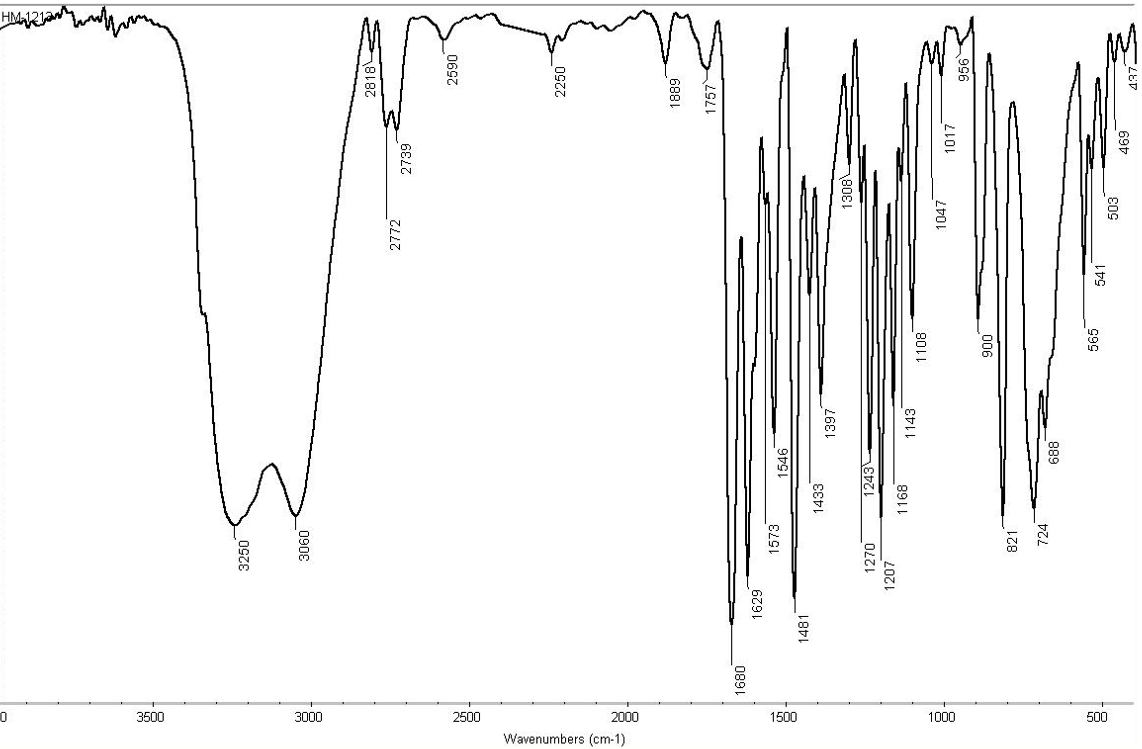
 **Fig. S21: IR spectrum of compound 4c**

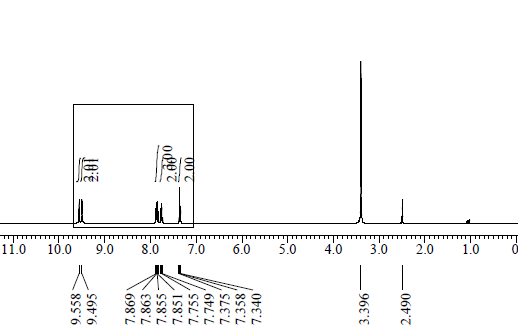


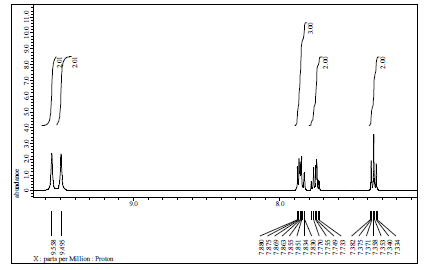


**Fig. S22: ^1^H-NMR spectrum of compound 4c**


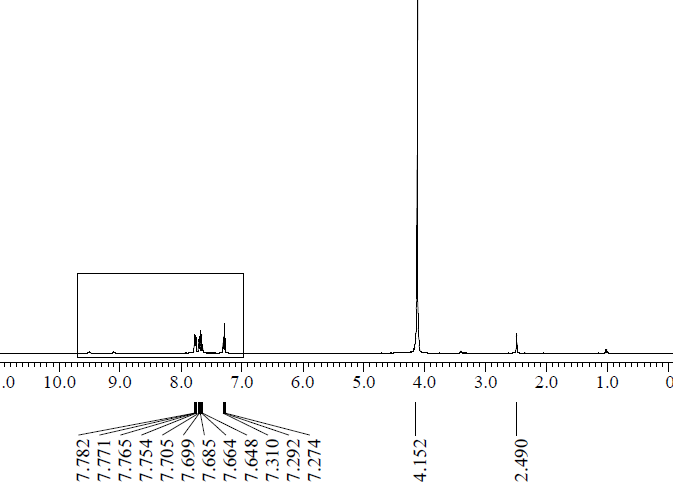

**Fig. S23: ^1^H-NMR (D_2_O) spectrum of compound 4c**

**
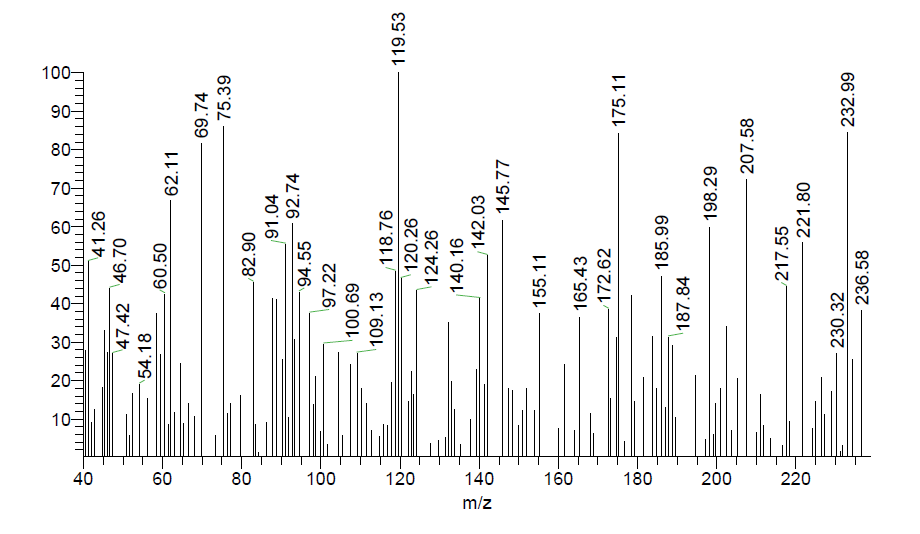
**

**Fig. S24: Mass spectrum of compound 4c**

**
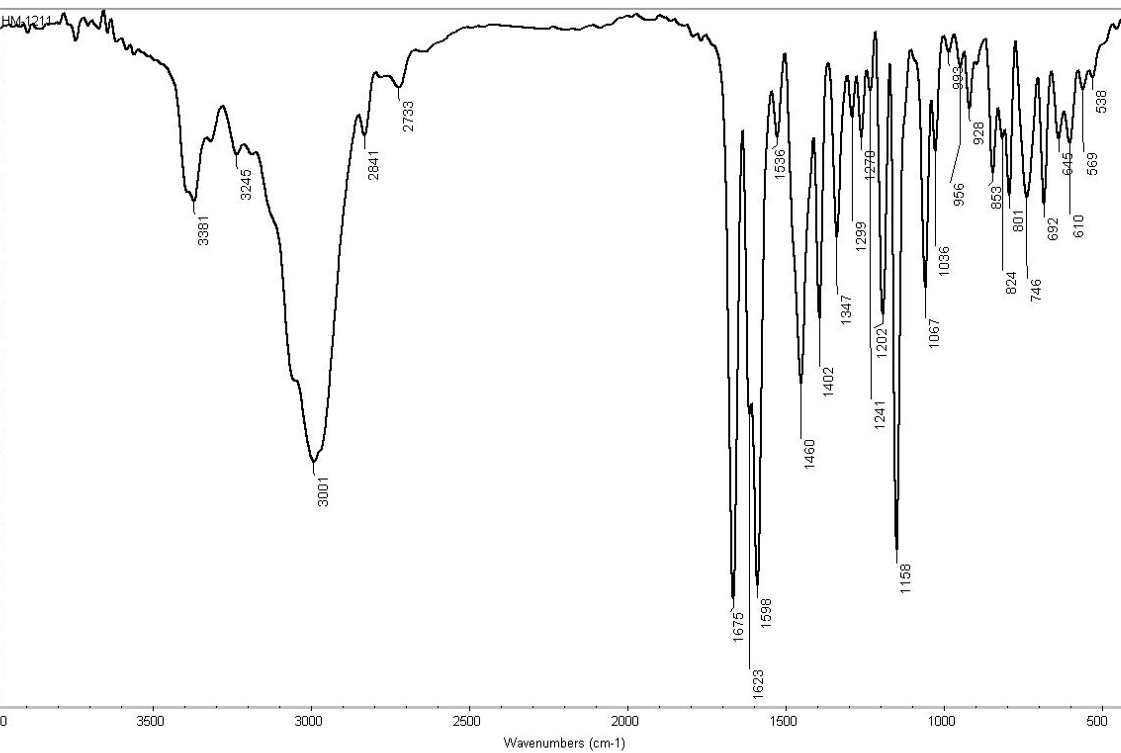
**

**Fig. S25: IR spectrum of compound 4d**

| **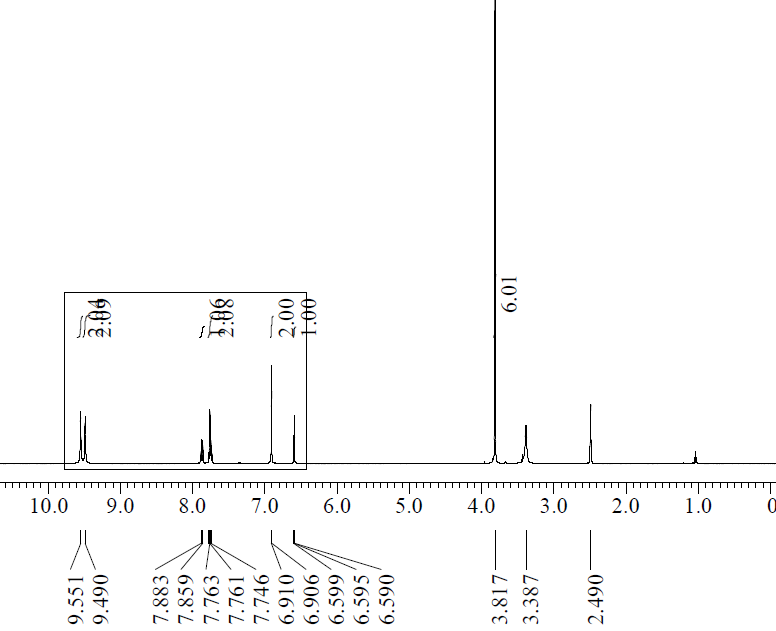**  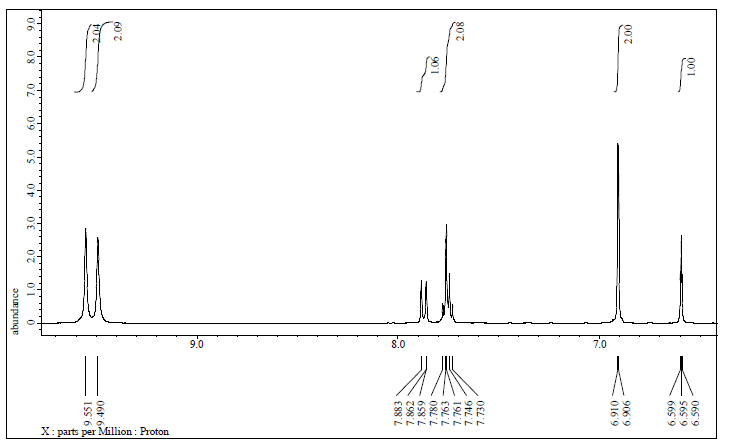   |
| --- |

**Fig. S26: ^1^H-NMR spectrum of compound 4d**


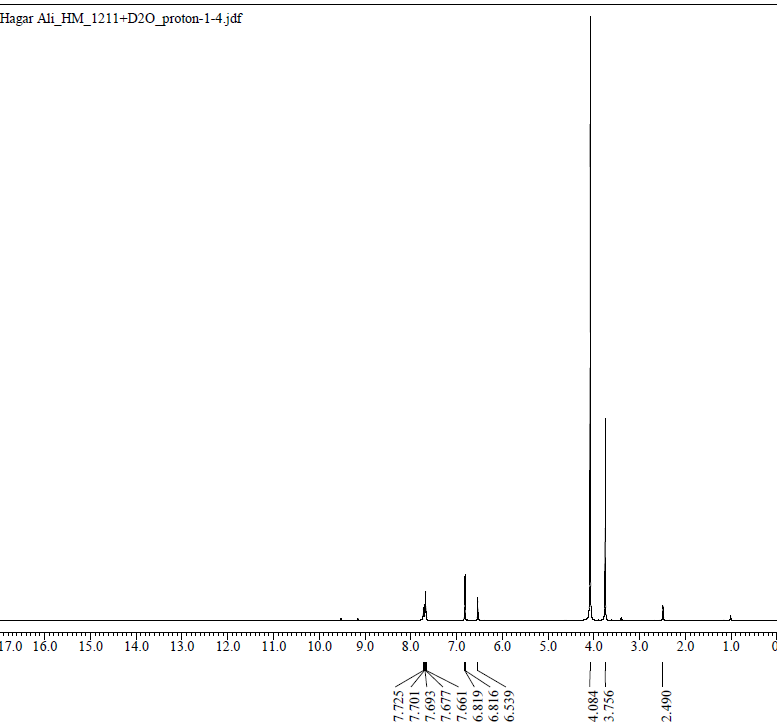

**Fig. S27: ^1^H-NMR (D_2_O) spectrum of compound 4d**


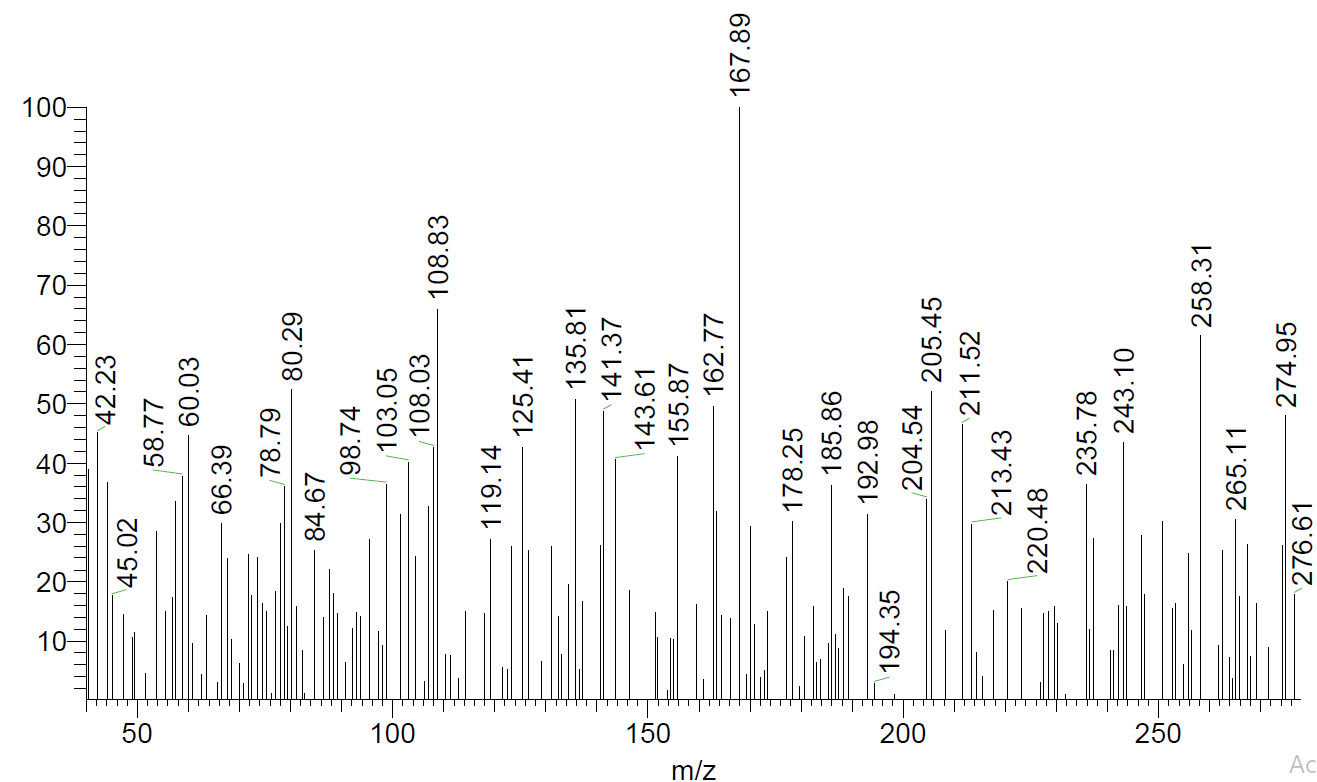

**Fig. S28: Mass spectrum of compound 4d**


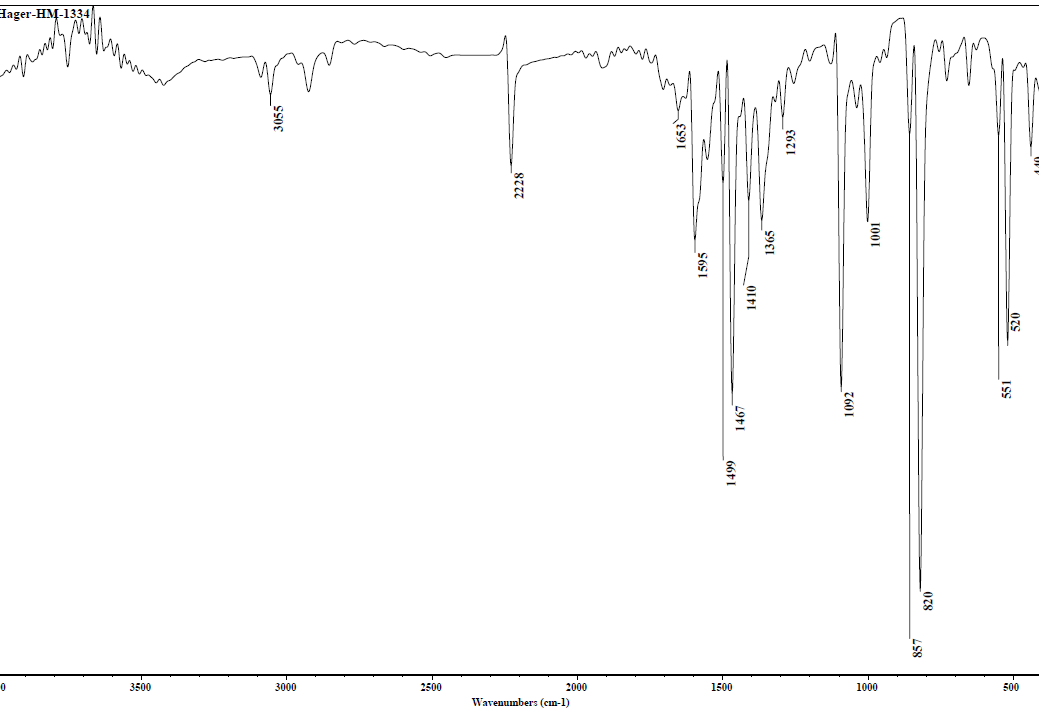

**Fig. S29: IR spectrum of compound 7a**

**
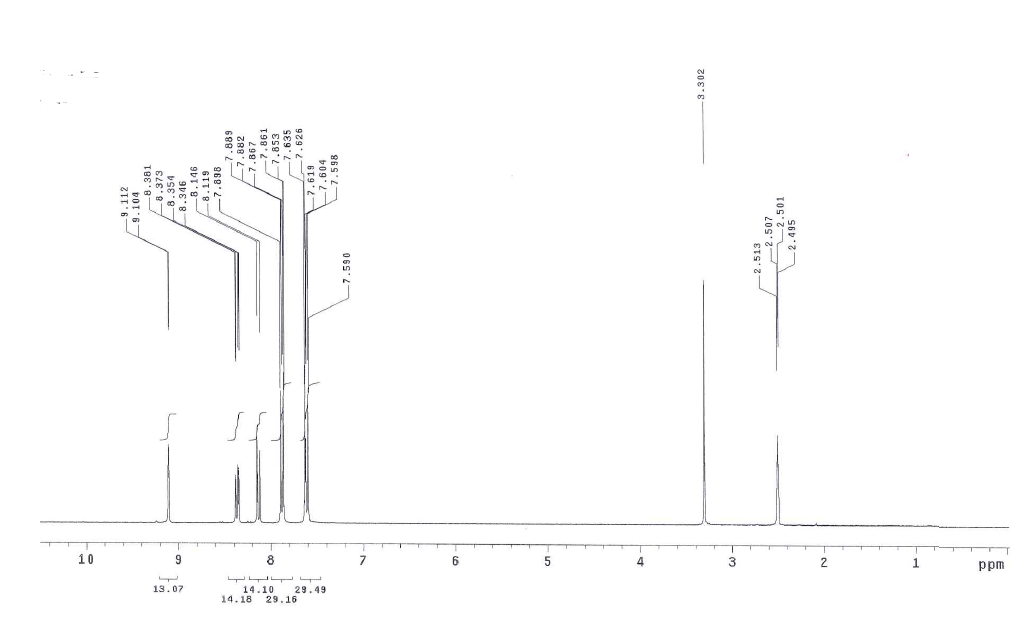
**

**Fig. S30: ^1^H-NMR spectrum of compound 7a**

| **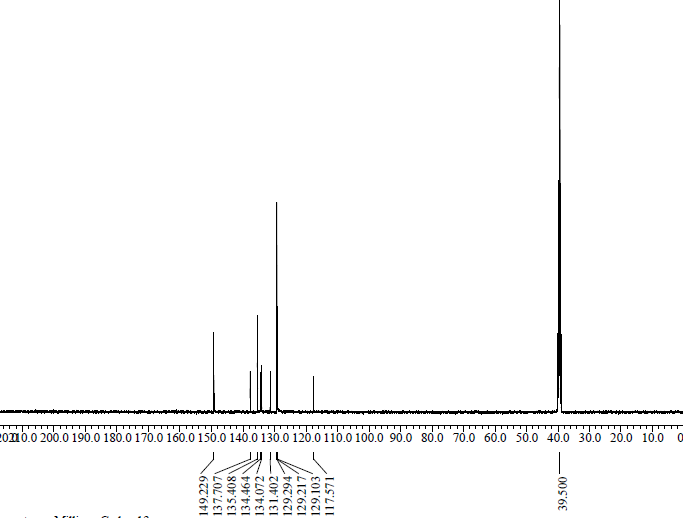**   |
| --- |

**^13^C-NMR spectrum of compound 7a**  **Fig. S31:**

**
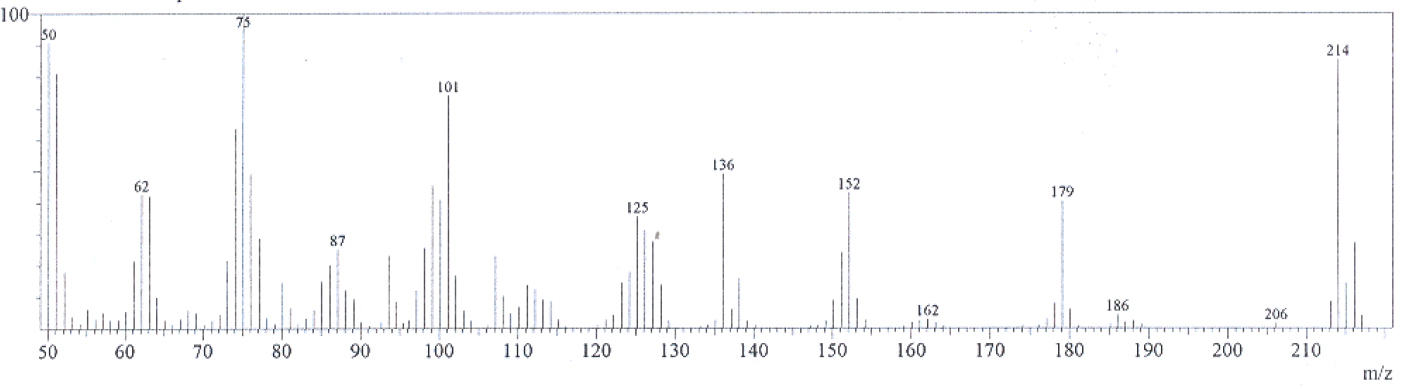
**

**Fig. S32: Mass spectrum of compound 7a**


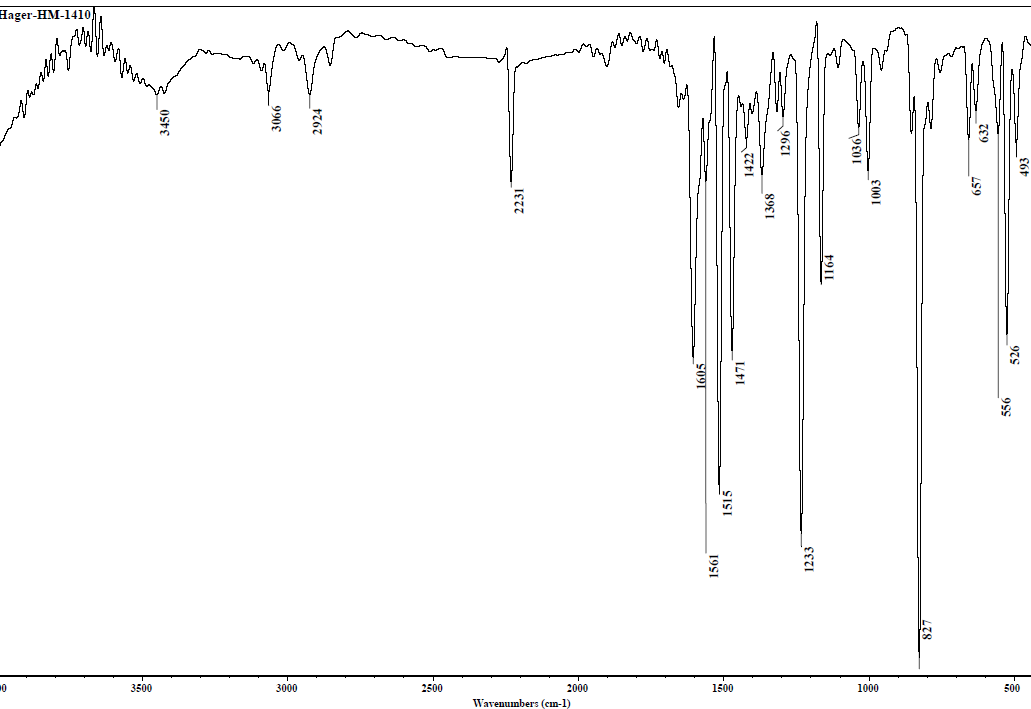

**Fig. S33: IR spectrum of compound 7b**


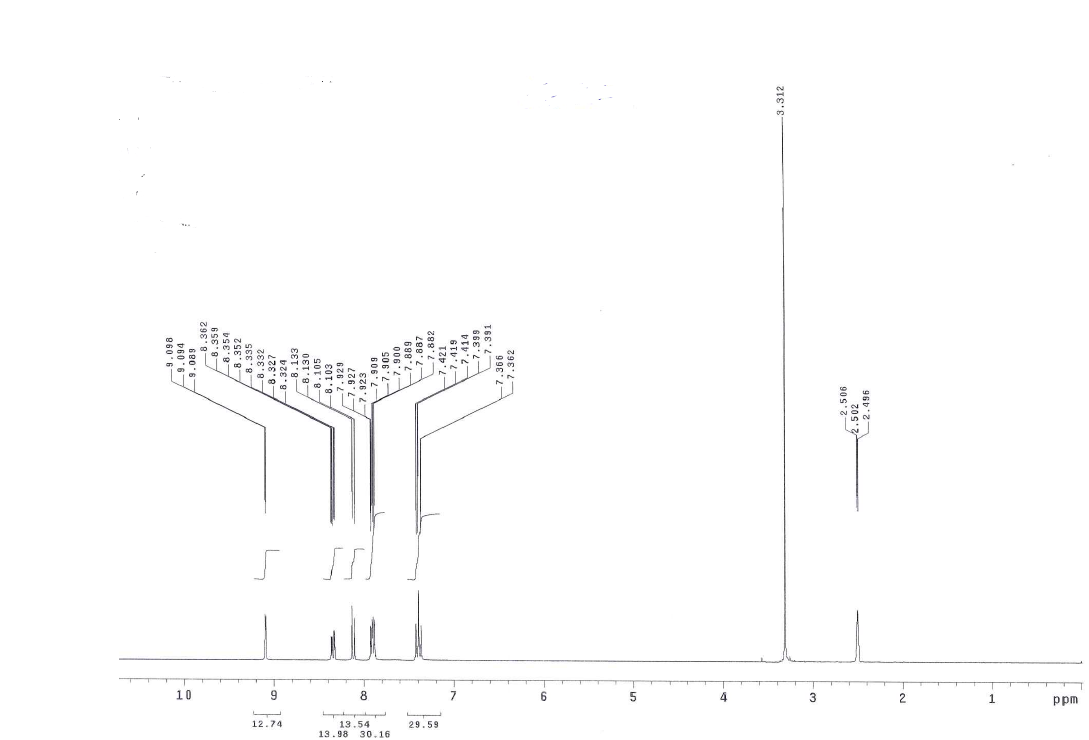

**^1^H-NMR spectrum of compound 7b** **Fig. S34:**


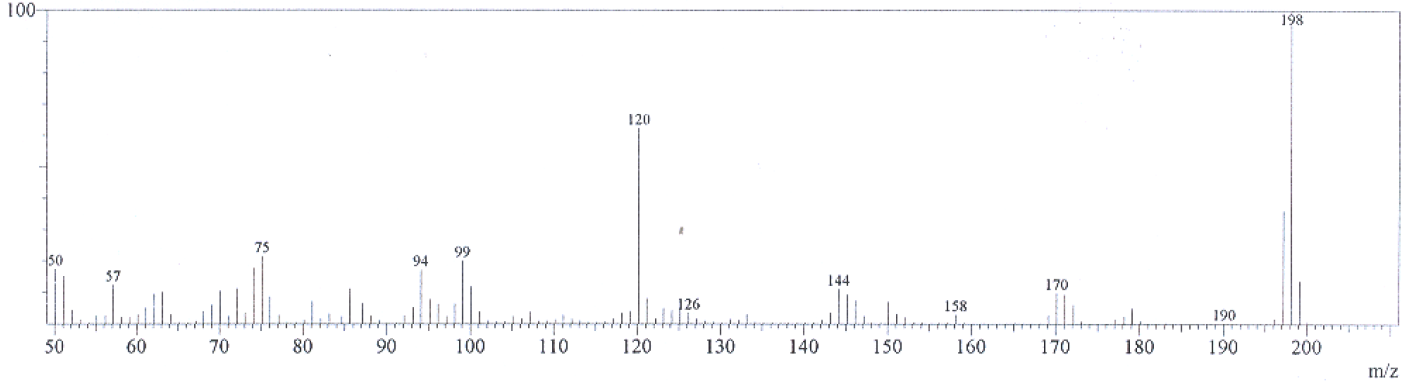

**Fig. S35: Mass spectrum of compound 7b**


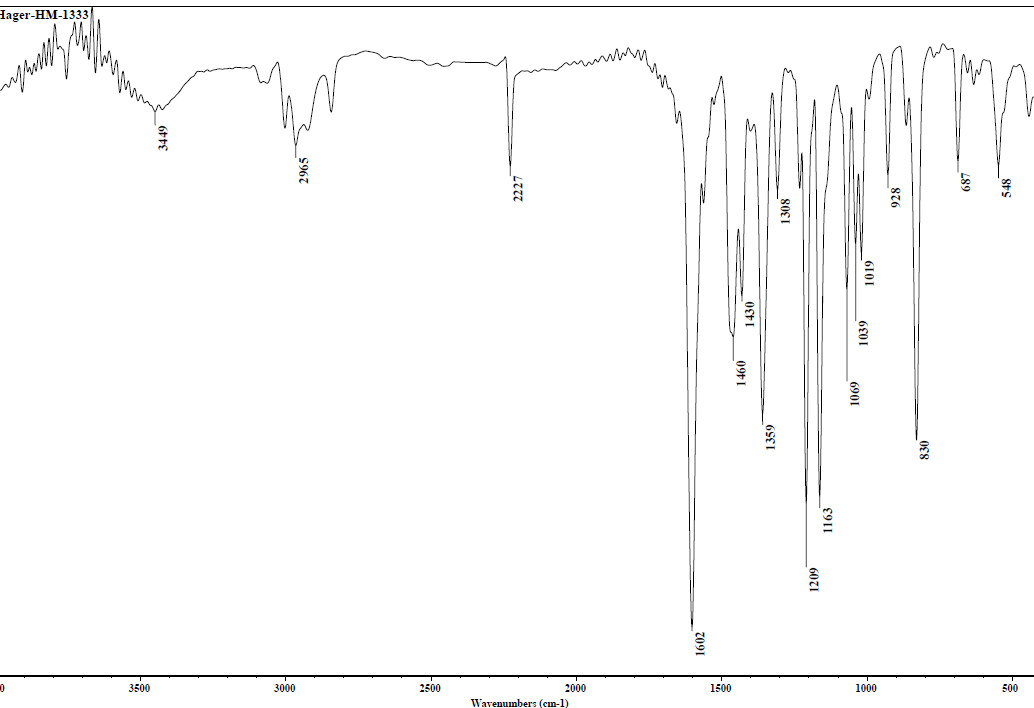

**Fig. S36: IR spectrum of compound 7c**


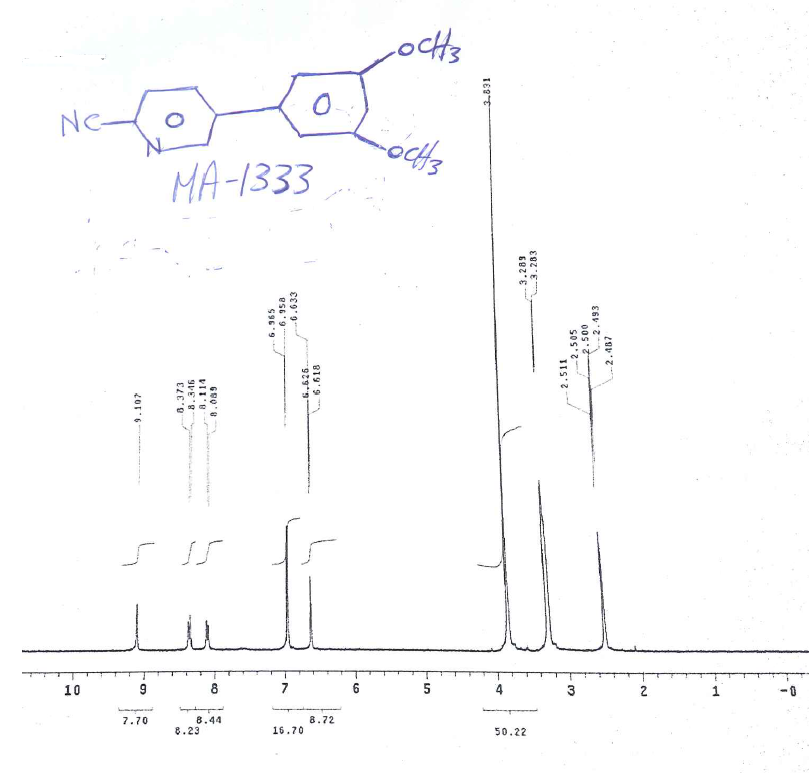

**^1^H-NMR spectrum of compound 7c Fig. S37:**

| **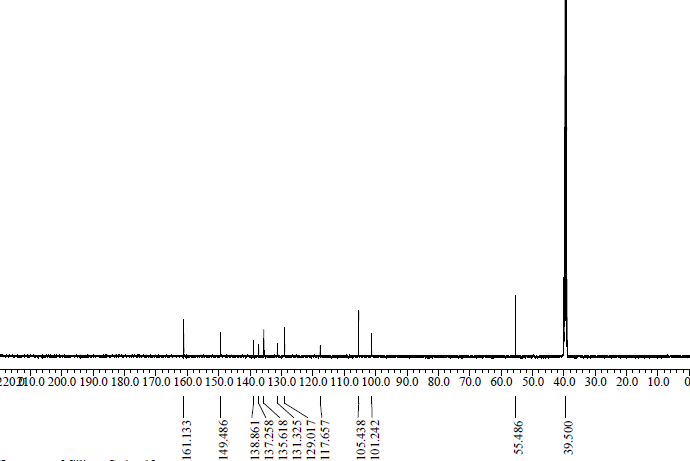**   |
| --- |

**Fig. S38: ^13^C-NMR spectrum of compound 7c**

**
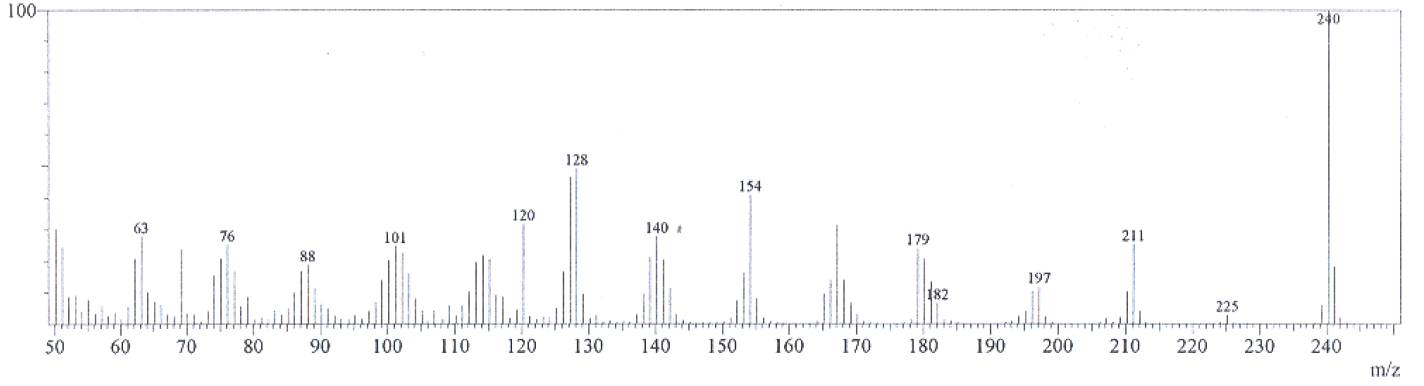
**

**Fig. S39: Mass spectrum of compound 7c**


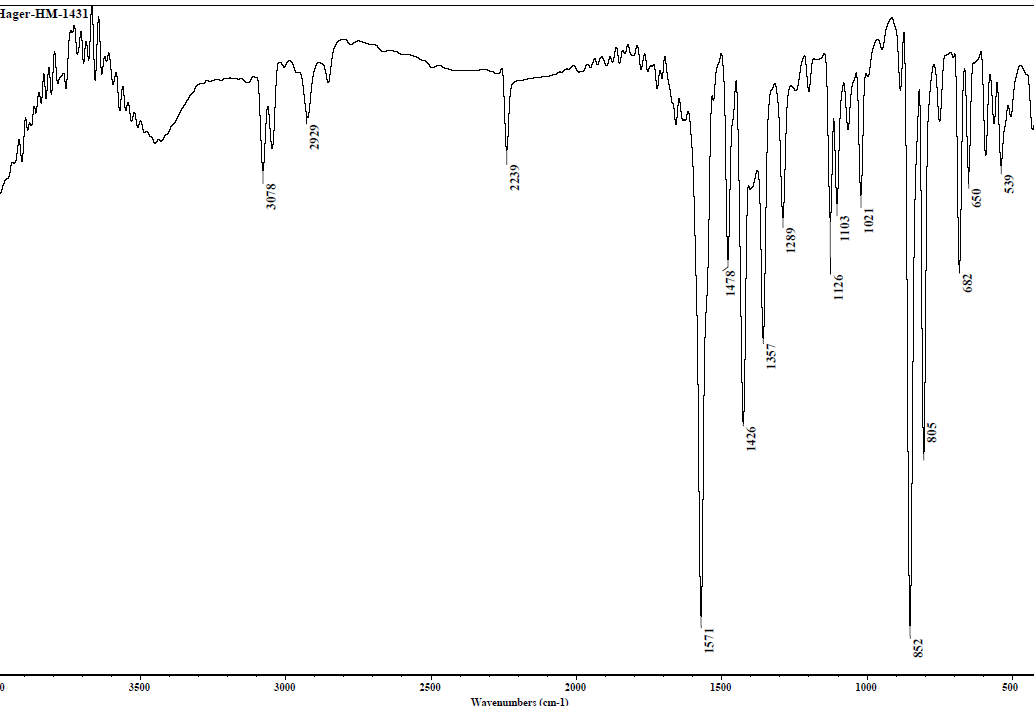

**Fig. S40: IR spectrum of compound 7d**

**
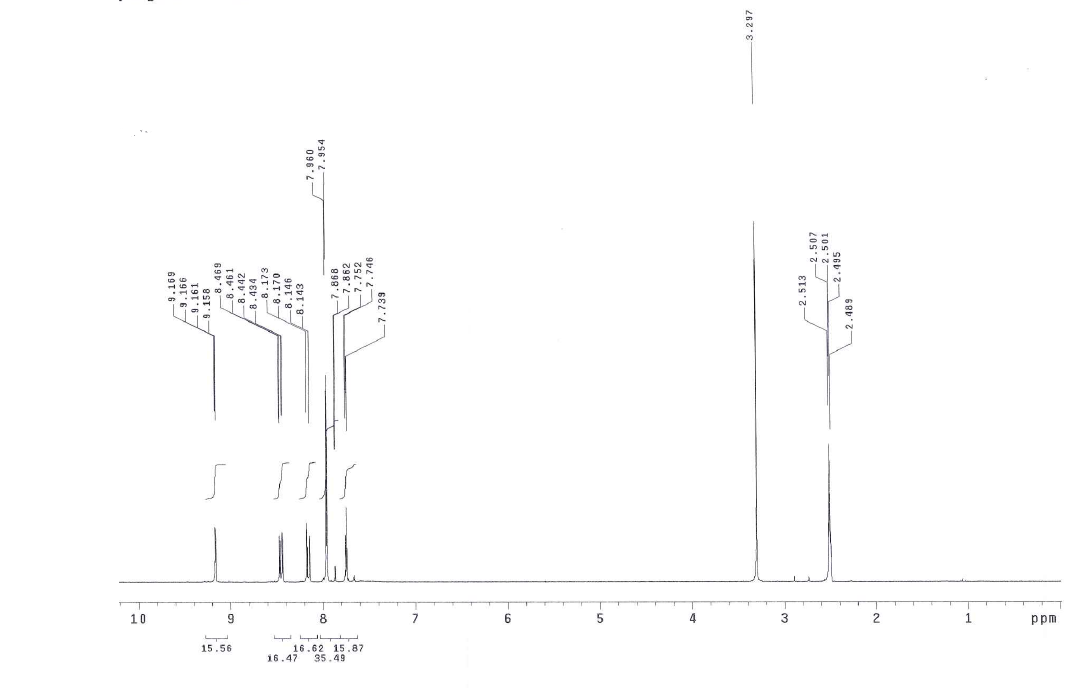
**

**Fig. S41: ^1^H-NMR spectrum of compound 7d**

**
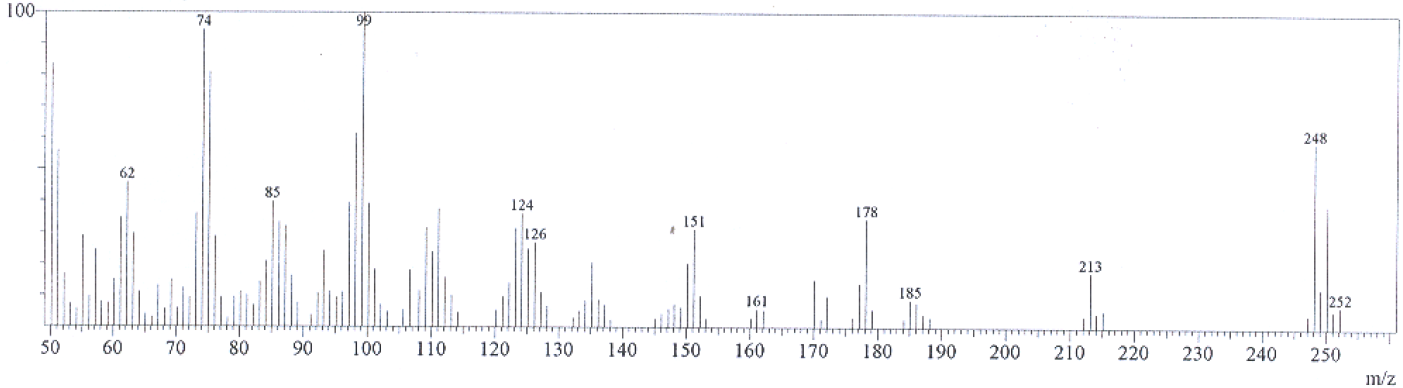
**

**Fig. S42: Mass spectrum of compound 7d**


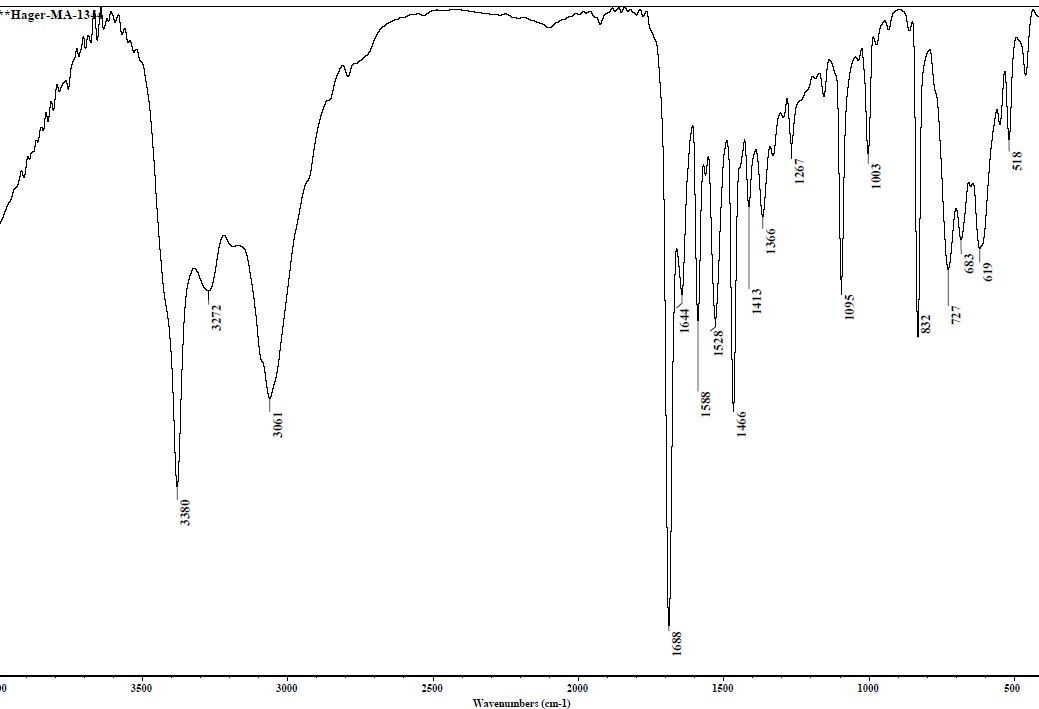

**Fig. S43: IR spectrum of compound 8a**

**
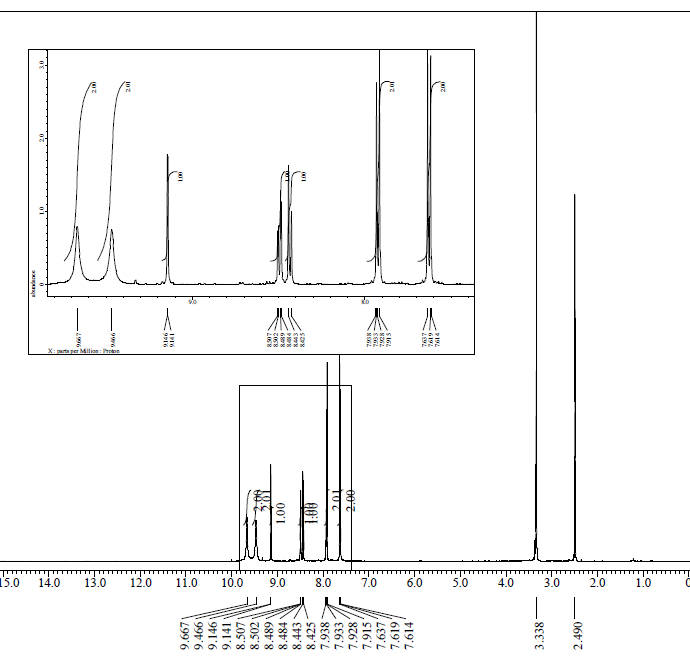
**

**Fig. S44: ^1^H-NMR spectrum of compound 8a**

**
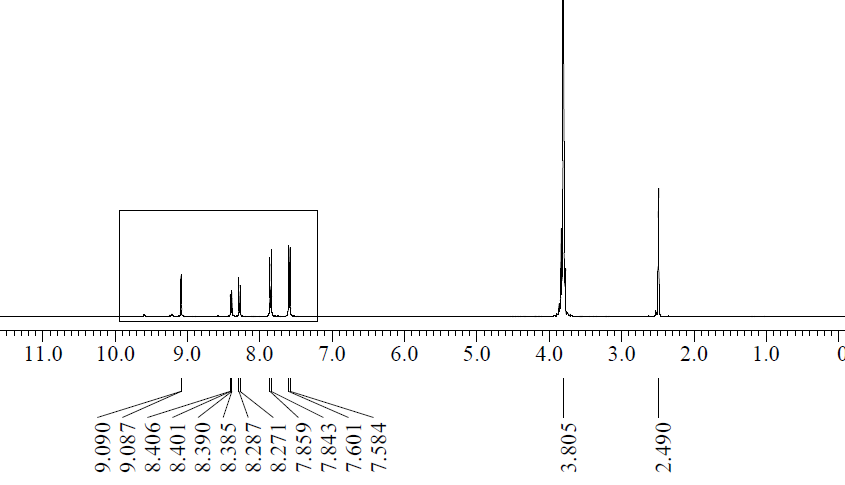
**

**^1^H-NMR (D_2_O) spectrum of compound 8a**  **Fig. S45:**


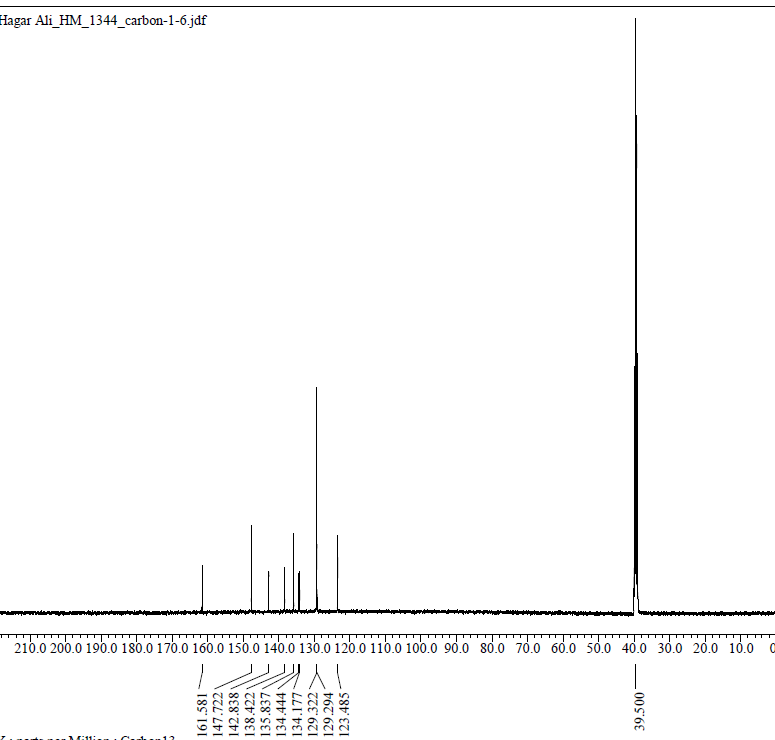

**Fig. S46: ^13^C-NMR spectrum of compound 8a**

**
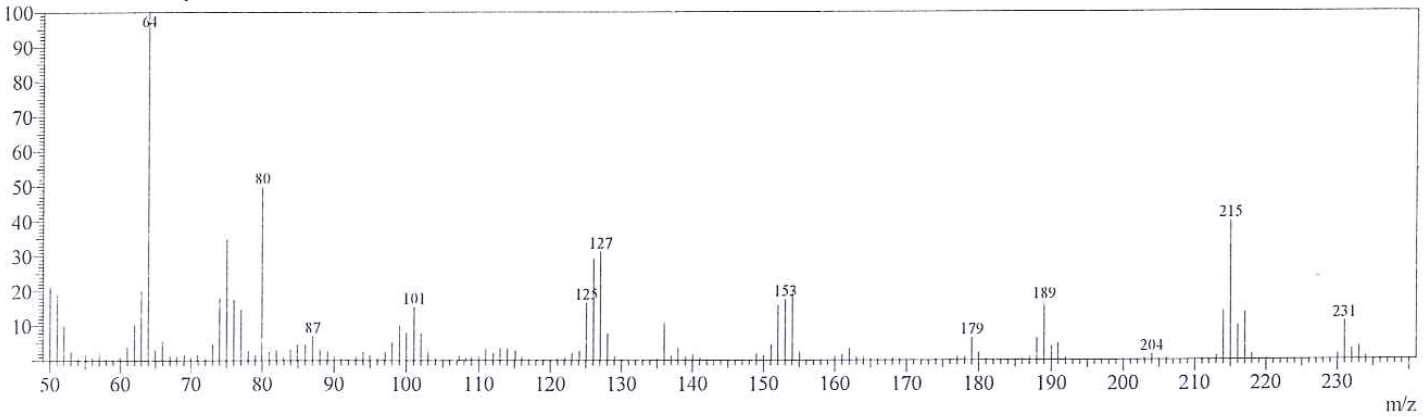
**

**Fig. S47: Mass spectrum of compound 8a**


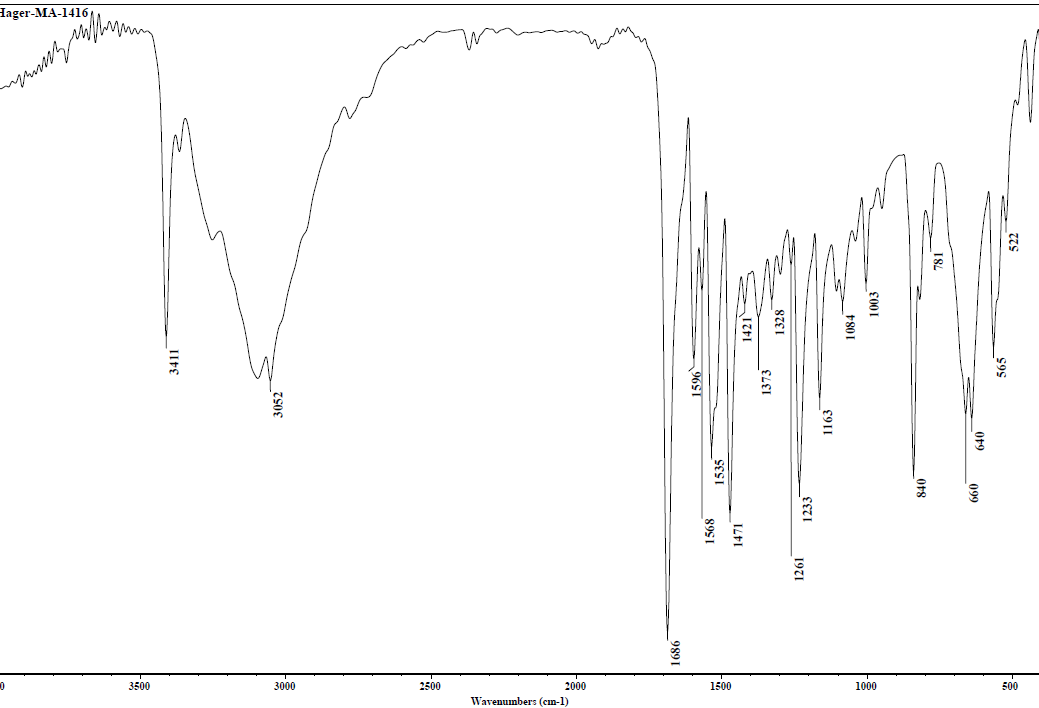

**Fig. S48: IR spectrum compound 8b**

**
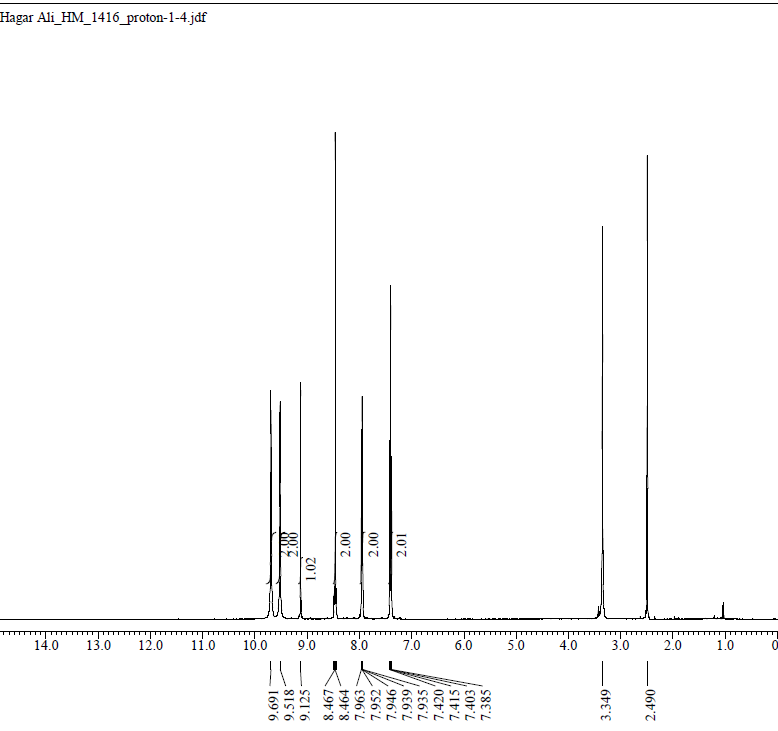
**

**Fig. S49: ^1^H-NMR spectrum of compound 8b**


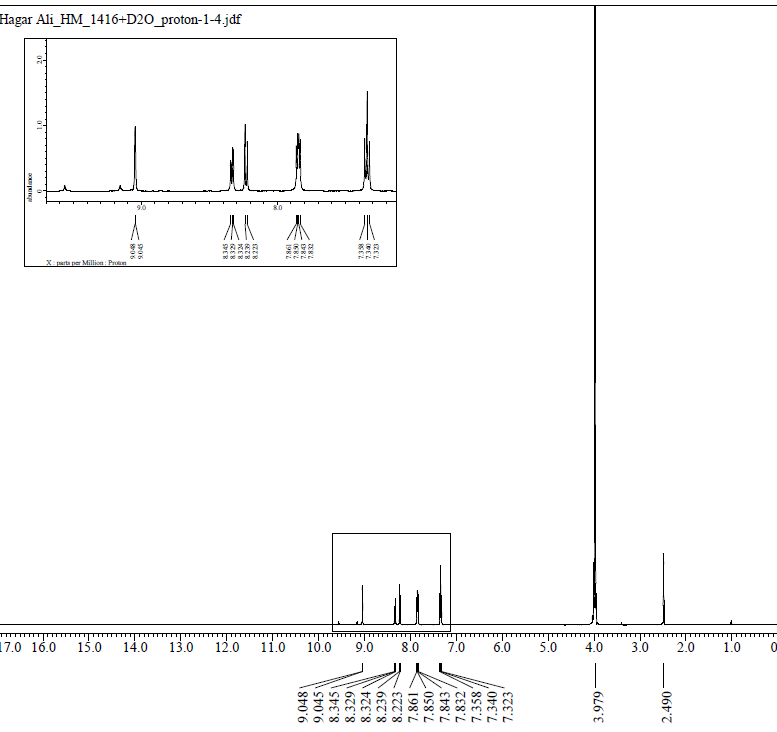

**Fig. S50: ^1^H-NMR (D_2_O) spectrum of compound 8b**

| 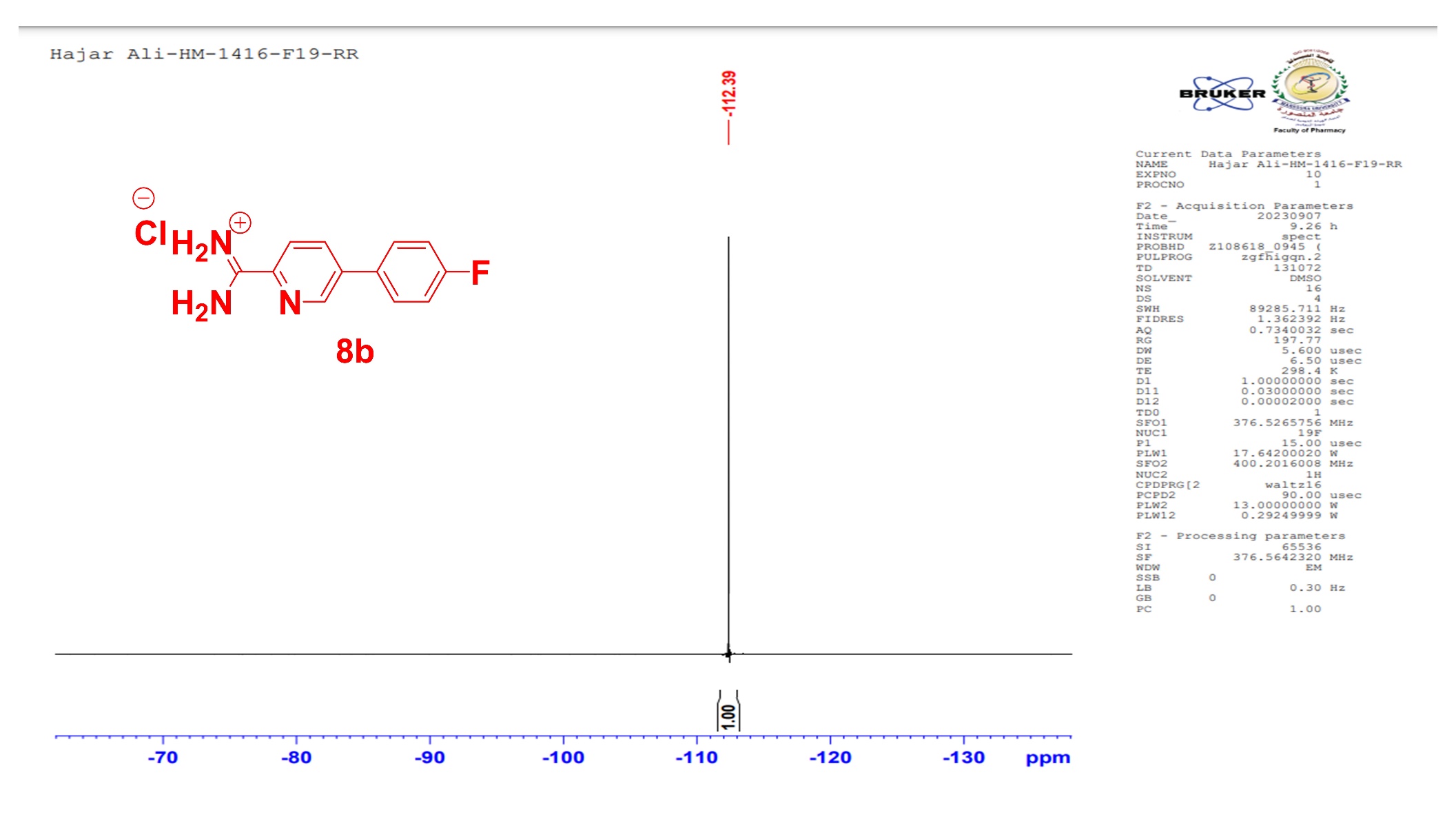 |
| --- |

**Fig. S51: ^19^F-NMR spectrum of compound** **8b**


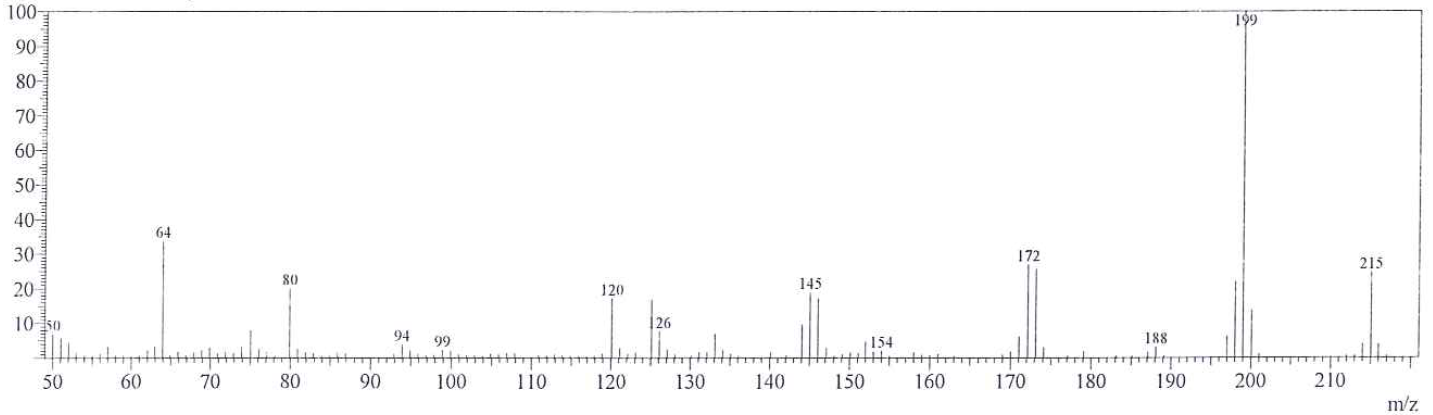

**Fig. S52: Mass** **spectrum of compound 8b**


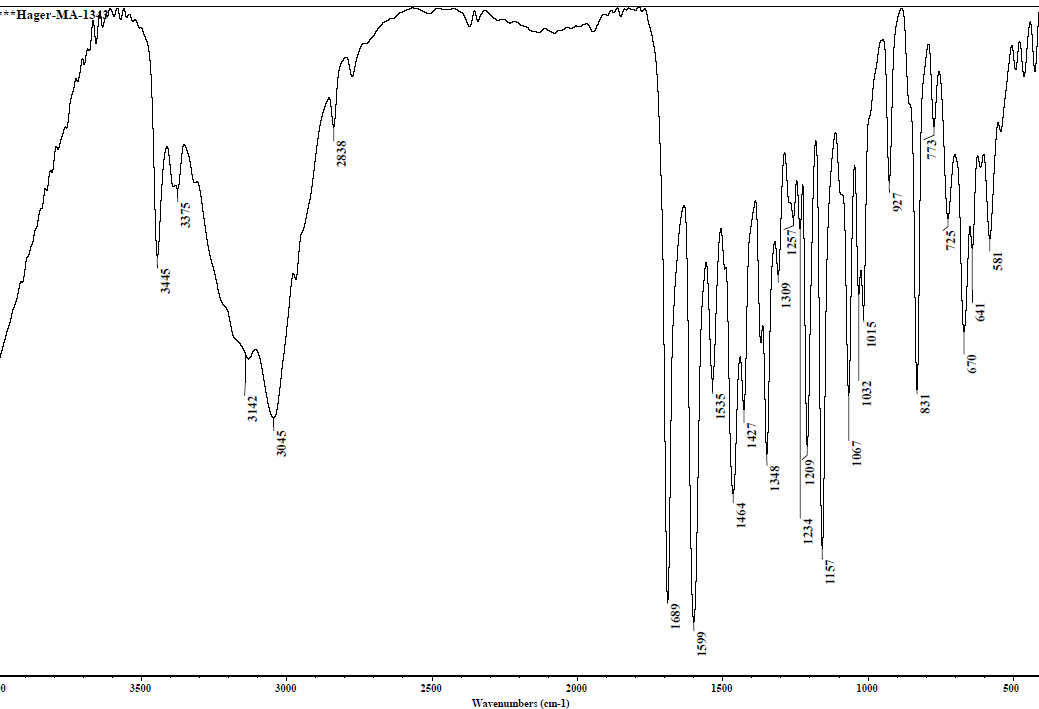

**Fig. S53: IR spectrum of compound 8c**


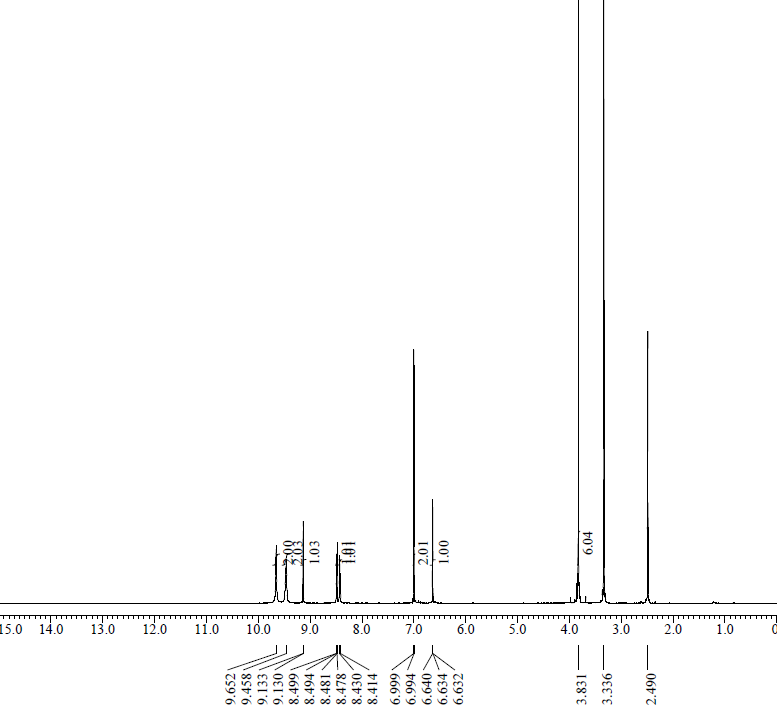

**^1^H-NMR spectrum of compound 8c**  **Fig. S54:**


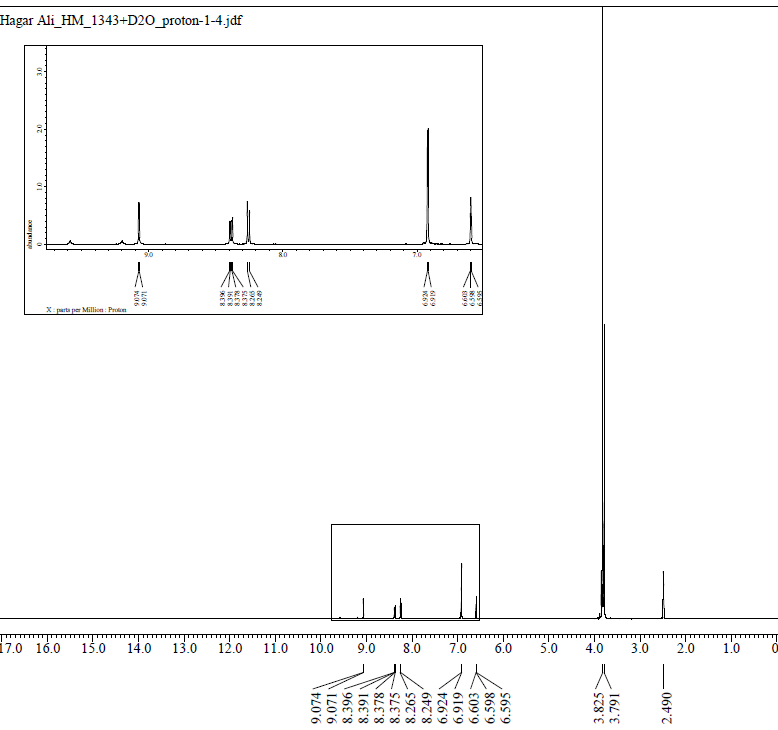

**Fig. S55: ^1^H-NMR (D_2_O) spectrum of compound 8c**


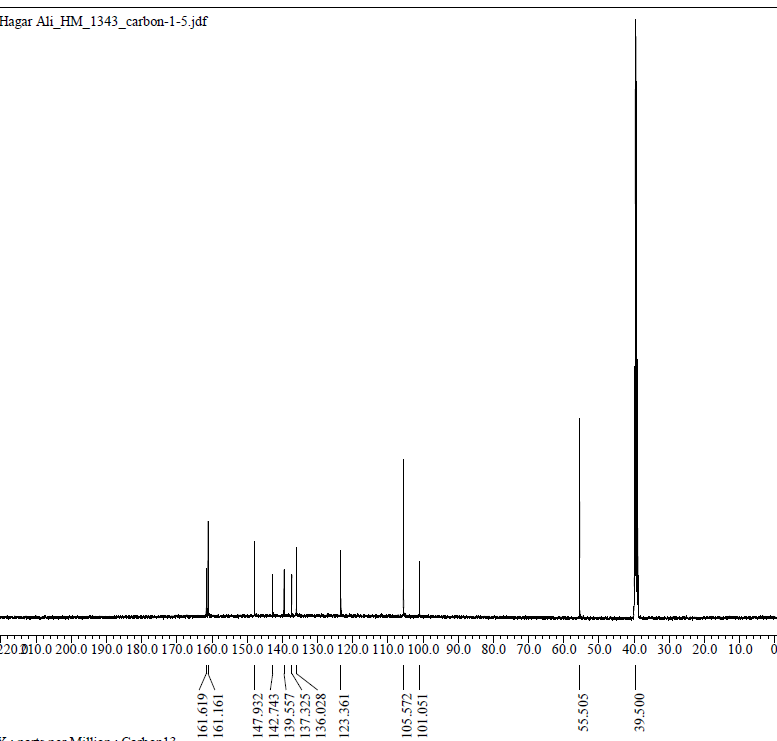

**Fig. S56: ^13^C-NMR spectrum of compound** **8c**


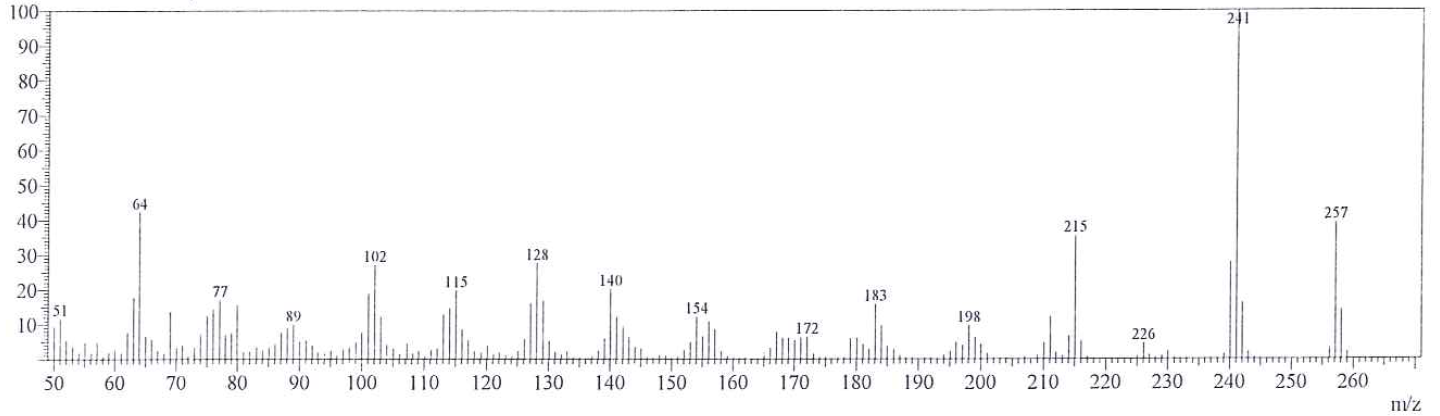

**Fig. S57: Mass spectrum of compound 8c**


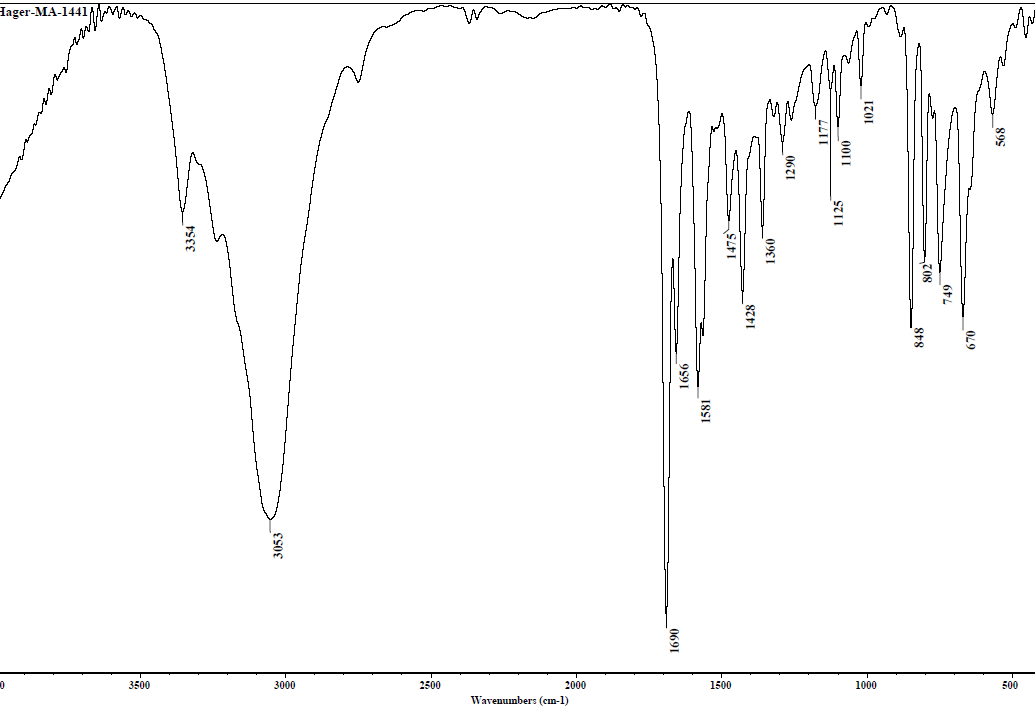

**Fig. S58: IR spectrum of compound 8d**

**
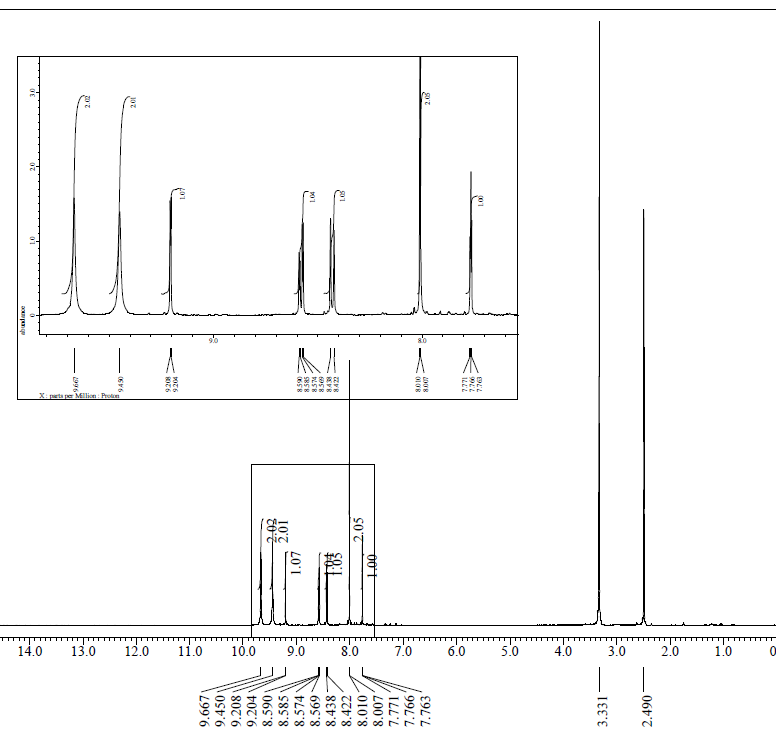
**

**Fig. S59: ^1^H-NMR spectrum of compound 8d**


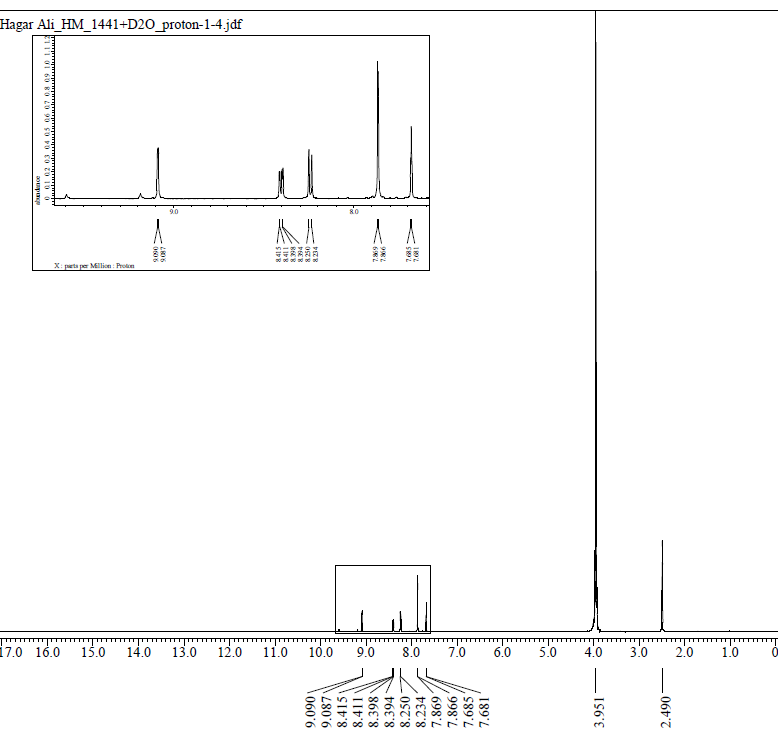

**Fig. S60: ^1^H-NMR (D_2_O) spectrum of compound 8d**


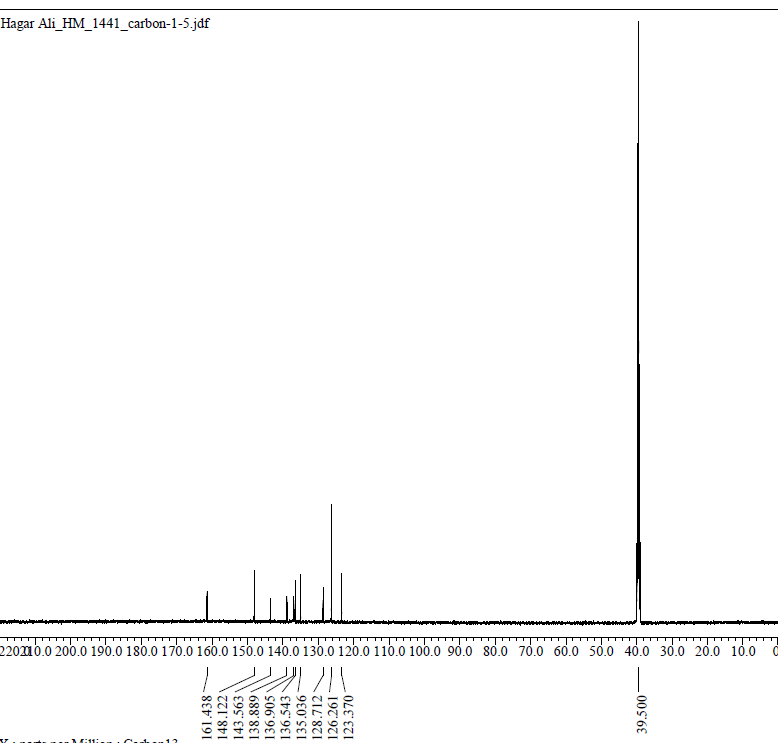

**^13^C-NMR spectrum of compound** **8d Fig. S61:**


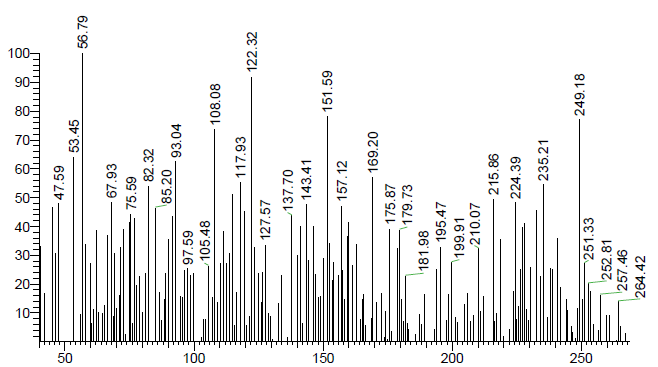

**Fig. S62: Mass spectrum of compound 8d**

**General:**

Melting points (uncorrected) were measured using a Gallenkamp melting-point apparatus and were uncorrected. The reaction mixture was monitored by using thin-layer chromatography (TLC) which was made on silica gel 60 F_254_ precoated aluminum sheets and visualized under ultraviolet (UV) light. Also, Infrared (IR) spectra were recorded using KBr wafer technique on a Thermo scientific Nicolet iS10 FT-IR Spectrometer. A JEOL 500 *MHz*, a Bruker 400 *MHz* & a Varian Unity Plus 300 *MHz* spectrometers were used for recording ^1^H- NMR, ^13^C-NMR and ^19^F-NMR spectra as the chemical shifts (*δ*) were measured in parts per million (ppm) relative to the used CDCl_3_ or DMSO-*d_6_* as solvents and self-internal standard. In case of cationic amidines NH_2_ & ^+^NH_2_ of cationic group were exchangeable with D_2_O. Electron impact mass spectra were determined at 70 eV on Varian MAT 311Kratos instrument (Micro-analytical center, Faculty of Science, Cairo University. All chemicals and solvents were used as received from Sigma Aldrich and Fisher scientific companies.
